# New insights into QTNs and potential candidate genes governing rice yield via a multi-model genome-wide association study

**DOI:** 10.1186/s12870-024-04810-5

**Published:** 2024-02-20

**Authors:** Supriya Sachdeva, Rakesh Singh, Avantika Maurya, Vikas K Singh, Uma Maheshwar Singh, Arvind Kumar, Gyanendra Pratap Singh

**Affiliations:** 1 Division of Genomic Resources, ICAR-NBPGR, Pusa, New Delhi, India; 2 International Rice Research Institute (IRRI), South Asia Hub, ICRISAT, Hyderabad, India; 3 International Rice Research Institute (IRRI), South Asia Regional Centre (ISARC), Varanasi, India; 4 International Crops Research Institute for the Semi-Arid Tropics, Patancheru, Telangana India; 5 ICAR-National Bureau of Plant Genetic Resources, Pusa, New Delhi, India

**Keywords:** Multi-model GWAS, Quantitative trait nucleotides, Superior alleles, Yield, Ontology, Enrichment analysis, Candidate genes, *Oryza sativa* L, Networks

## Abstract

**Background:**

Rice (*Oryza sativa* L.) is one of the globally important staple food crops, and yield-related traits are prerequisites for improved breeding efficiency in rice. Here, we used six different genome-wide association study (GWAS) models for 198 accessions, with 553,229 single nucleotide markers (SNPs) to identify the quantitative trait nucleotides (QTNs) and candidate genes (CGs) governing rice yield.

**Results:**

Amongst the 73 different QTNs in total, 24 were co-localized with already reported QTLs or loci in previous mapping studies. We obtained fifteen significant QTNs, pathway analysis revealed 10 potential candidates within 100kb of these QTNs that are predicted to govern plant height, days to flowering, and plot yield in rice. Based on their superior allelic information in 20 elite and 6 inferior genotypes, we found a higher percentage of superior alleles in the elite genotypes in comparison to inferior genotypes. Further, we implemented expression analysis and enrichment analysis enabling the identification of 73 candidate genes and 25 homologues of *Arabidopsis*, 19 of which might regulate rice yield traits. Of these candidate genes, 40 CGs were found to be enriched in 60 GO terms of the studied traits for instance, positive regulator metabolic process (GO:0010929), intracellular part (GO:0031090), and nucleic acid binding (GO:0090079). Haplotype and phenotypic variation analysis confirmed that LOC_OS09G15770, LOC_OS02G36710 and LOC_OS02G17520 are key candidates associated with rice yield.

**Conclusions:**

Overall, we foresee that the QTNs, putative candidates elucidated in the study could summarize the polygenic regulatory networks controlling rice yield and be useful for breeding high-yielding varieties.

**Supplementary Information:**

The online version contains supplementary material available at 10.1186/s12870-024-04810-5.

## Background

Rice is one of the major cereal crops that feeds over half of the human population and has been grown mainly in Asian countries for more than hundred decades [1–3]. Increasing yield potential has been a long-term objective in rice breeding and is imperative to overcome the global food crisis [4–9]. Rice yield is the most targeted complex trait that is a direct function of multiple factors including number and size of grains, productive tillers per plant, plant count per unit area, size of panicles and plant height [2, 7, 10–12] thus, the aforesaid component traits are prerequisite for achieving the desired yield in rice. This warrants the dissection of yield-related quantitative traits, discovery of the novel genetic factors and elucidate their molecular basis to meet the increased global rice demand.

New developments in genomic technologies, molecular markers, wealth of germplasm collections and genome information available on public portals have greatly facilitated deciphering of causative loci of complex traits for use in rice breeding [4, 13–19]. Essentially, Genome-wide association study (GWAS) has been evidenced to be a reliable approach for localization of genetic factors conferring quantitative traits to a narrow region [20–30] and has been successfully employed in different crop species such as rice, wheat, barley, chickpea, field pea, mungbean, linseed, flax and soybean [31–38]. Earlier genome studies reveal making use of dense coverage single nucleotide polymorphism markers in association analysis facilitates identification of functional variations governing yield related traits [39–45] connecting the functional implications of high throughput GWAS results and the known grain yield genes has become a standard approach for boosting rice productivity that may also be appropriate for other crop plants [46].

Extensive researches on identification of genetic variants for economically important traits have been performed using single-locus (SL) methodologies including MLM [47] and CMLM [48] that have limited quantitative trait nucleotides (QTNs) detection ability due to their polygenic nature and conservative Bonferroni correction factor involved [49, 50]. Multiple multi-dimensional/locus (ML) methods of genome analysis *viz.,* mrMLM [46], MLMM [51], FASTmrEMMA [50], FASTmrMLMM [52] were, therefore, proposed to estimate all the marker effects simultaneously with lesser running time, computational load and increased power accurately and identify the QTNs related to quantitative traits precisely yield related [12, 53–55]. Recent resequencing efforts at IRRI, Philippines also suggested harnessing germplasm diversity and novel alleles related to grain yield traits might play a vital role in rice breeding and improvement [6, 56, 57]. Furthermore, advances in functional genomics, proteomics and metabolome studies, and their integration into a systems approach might prove instrumental in designing new higher yielding rice varieties for the future and accomplish the goals of rice biologists [46, 58]. CG-based association strategy has been recommended as a promising approach to develop ‘tailored rice’ suiting the ever-increasing rice demands [6].

The overall objective of our multi-model GWAS study was to identify the QTNs and CGs associated with yield traits *viz.,* plant height, days to flowering and plot yield across the selected subset of 3000 re-sequenced genome panel which were phenotyped during kharif 2020 and kharif 2021. We discovered common QTNs for the three aforesaid yield traits identified by both single and multi-locus models. We then conducted gene mining for the CGs in close vicinity of these significant QTNs and identified elite accessions with superior alleles in the selected subset which might accelerate selection breeding for higher yielding rice varieties. Additionally, we combined the expression data online available and pathway analysis results to better understand the molecular basis of these quantitative traits and improve grain yield in rice.

## Material and methods

**Fig. 1:**
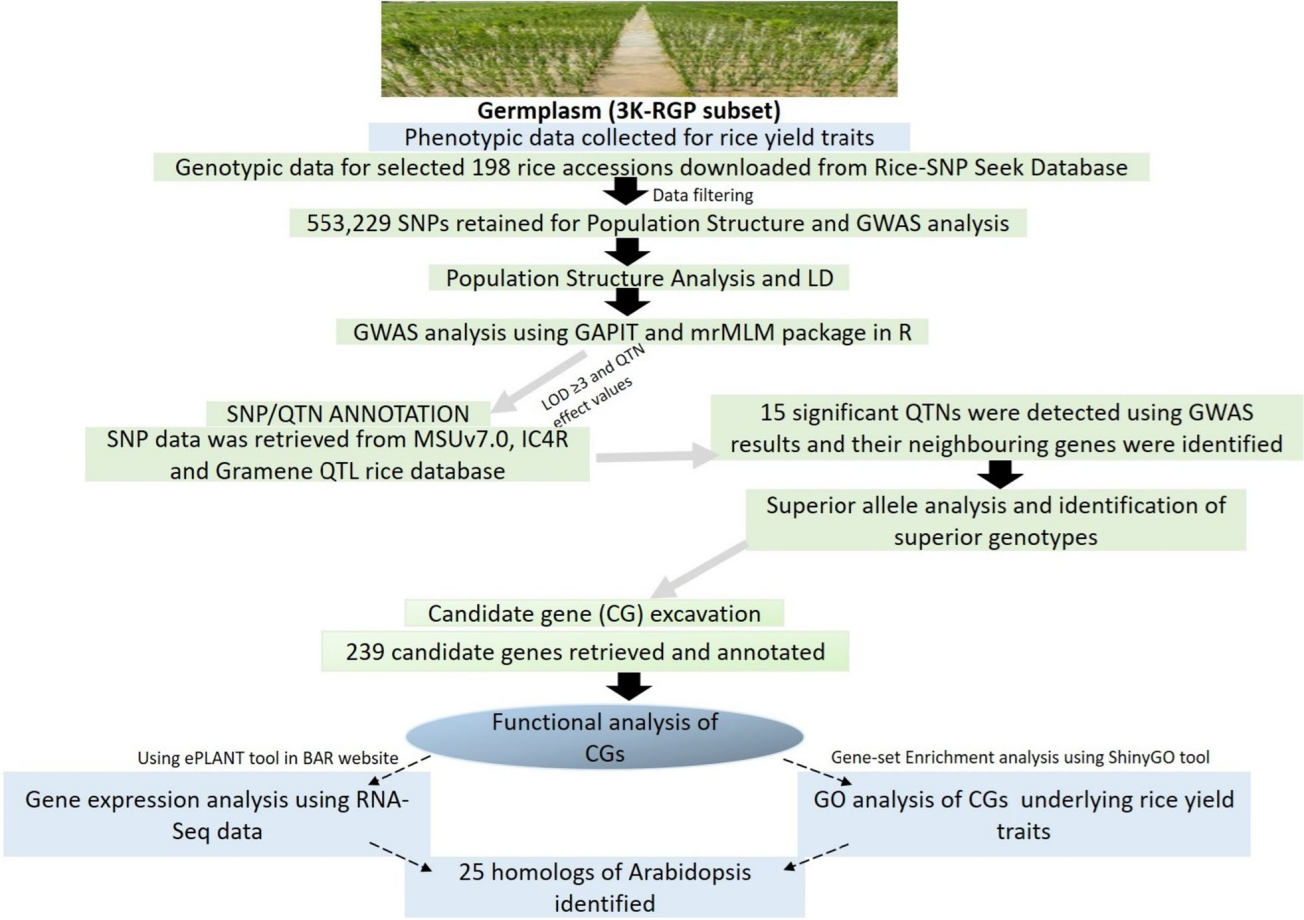
Schematic representation of the methodology followed in our study

A simplified flow chart has been illustrated and presented (Fig. 1) to better understand the methodology adopted in execution of our multi-model GWAS study, and the steps implemented are as ensued:

### Material, field trial and measurement of grain yield traits

**Fig. 2:**
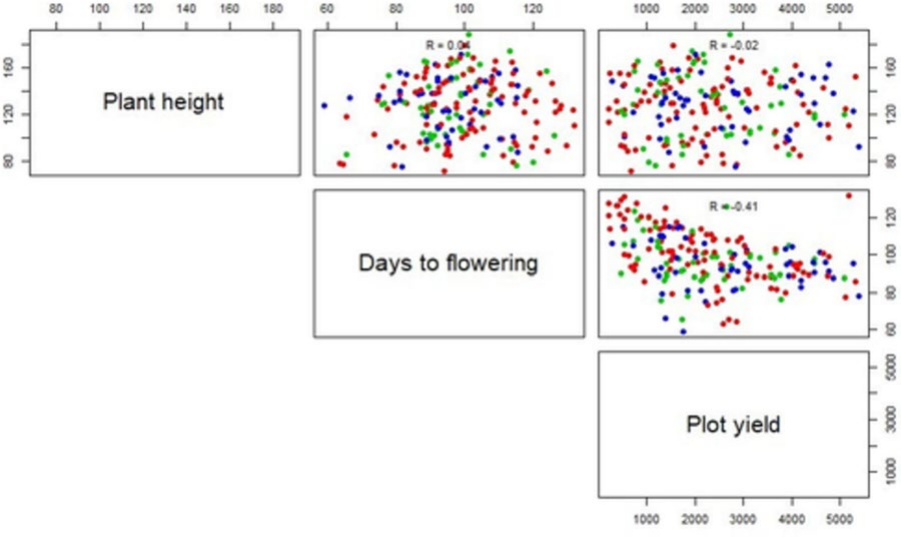
Distribution of three yield traits in rice and Pearson’s coefficients. Color gradients depict range of trait values. Blue, green and red dots indicate lower, moderate and high values of grain yield traits. Pl Ht, Plant height; DTF, Days to flowering; PYD, Plot yield

The rice subset of 196 accessions from 3000 genomes re-sequenced panel (https://doi.org/10.1186/2047-217X-3-7) collected from IRRI South Asia Regional Center (IRRI-SARC), Varanasi, India and 2 check varieties *viz.,* Pusa Basmati 1121 and Lalat were used as the materials in our study. These genotypes originating from 32 countries are presented in Table S1. The complete subset was planted in the ICAR-IARI farm, New Delhi, India during Kharif season of 2020 and 2021 with augmented RCB design in 7 blocks. The seeds were sown in a nursery bed and 21 days old seedlings were then replanted in puddled experimental plots in two rows with a distance of 20x15cms and four replications. Recommended farmer practices were followed for management of transplanted rice. Plant height(PlHt, cms), days to 50% flowering (DTF) and plot yield (PYD, kg/ha) was then recorded from five randomly selected healthy looking plants/plot as per the Rice Standard Evaluation System (http://www.knowledgebank.irri.org/images/docs/rice-standard-evaluation-system.pdf). All the three yield traits were visualized in R Studio, including the range, mean, standard deviation, coefficient of variation (CV), kurtosis and skewness (Table S2). Phenotypic correlations were also computed using the R package corrr (https://www.r-project.org/) (Fig. 2).

### SNP data genotyping, genetic diversity, structure analysis and LD

**Fig. 3:**
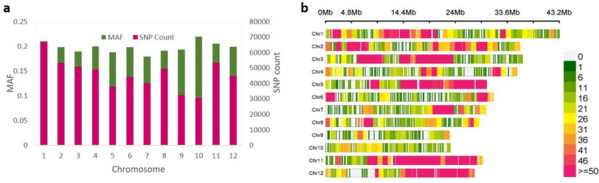
The distribution of SNP markers on 12 rice chromosomes. **a** Distribution of average MAF. **b** SNP markers density across different chromosomes

**Fig. 4:**
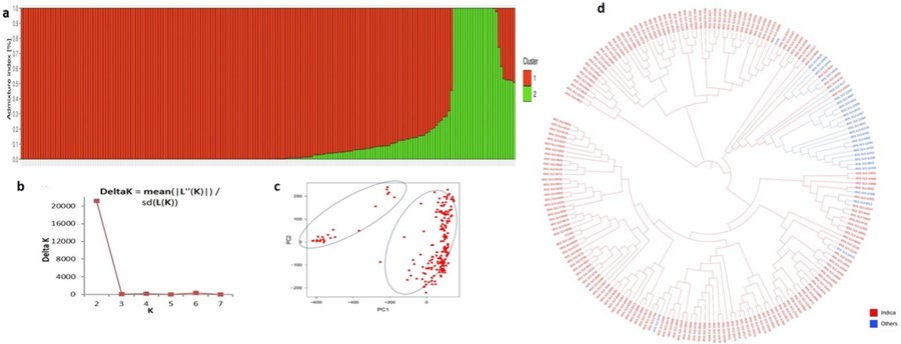
Population Structure analysis, principal components and neighbour joining tree analysis representing 198 rice accessions. **a** Population structure (K=2); the areas of the two colors (red and green) depict the two subpopulations. **b** Plot of ∆K calculated for K =2. **c** Distribution of selected accessions along the three PCs. PC1 and PC2 refer to the first and second principal components respectively. Dots denote each accession of the rice subset. **d** Neighbour joining tree with different colours represent the subpopulations identified in our study i.e. red and blue represent the indica subspecies and other subpopulations, respectively

The genotypic data for the selected accessions was downloaded from the online repository of variants of rice, the Rice SNP-seek database (http://snp-seek.irri.org/). Rice dataset comprising of 553,229 SNPs was obtained across the rice genome with a minor allele frequency (MAF) >5% and major allele frequency <95% for structure analysis and GWAS. Plots showing MAF and uniform SNP density distribution across the chromosomes illustrate suitability of the SNP dataset for genetic dissection of yield related traits in rice (Fig. 3a-b). Genetic locus-based diversity estimates, such as gene diversity (PiPerBP), minor allele frequency (MAF), expected number of polymorphic sites per nucleotide (ThetaPerBP), observed heterozygosity (H_o_) were calculated using TASSELv5.2.82 software (Table S3). The genetic distance matrix was then calculated, following the neighbour-joining method, and a phylogenetic tree was constructed, visualized using interactive tree of life (iTOL) software. Principal component analysis (PCA) was performed using R package prcomp to further validate the NJ results. Bayesian model-based structure analysis was executed in STRUCTUREv2.3.4 [59] wherein the supposed number of subpopulations ranged from 1 to 7 and with each K repeated three times. The burnin period of 100,000 iterations and 100,000 MCMC (Markov Chain Monte Carlo) period were implemented for each run and the number of subgroups were identified using the Evanno criterion [60]. The optimum K value was then determined using the STRUCTURE HARVERSTER [61] (Fig. 4). Tidyverse and ggplot2 package were used to determine the LD between each pair of SNPs and the trend of Linkage disequilibrium (LD) decay was assessed using the squared coefficient of correlation (r^2^) values of alleles and physical distance in Mb (Fig. S1).

### GWAS and favourable allele analysis

**Fig. 5:**
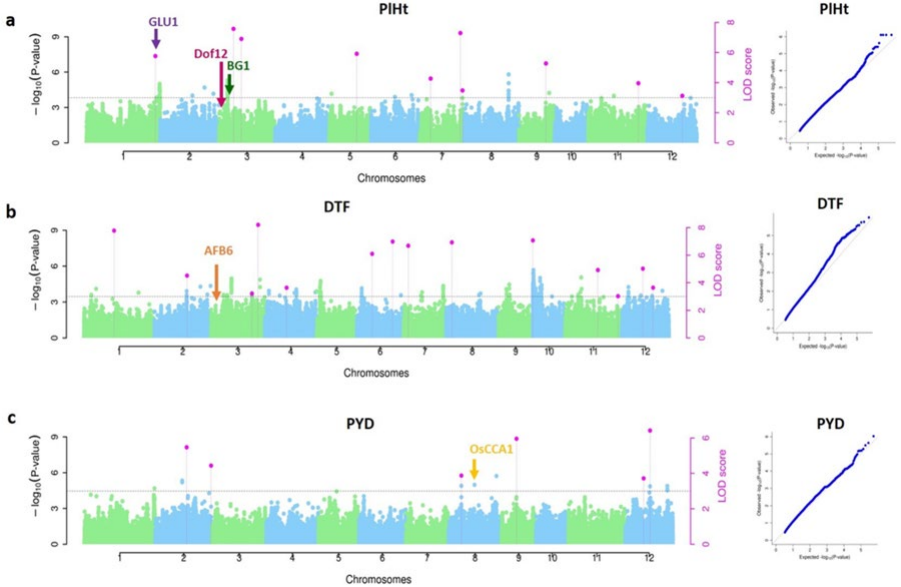
Manhattan plots and quantile-quantile plots for PlHt, DTF and PYD using three multi-locus models. The horizontal dotted line indicates the threshold LOD score ≥3. The dots above the threshold value represent the significant QTNs at different rice chromosomes, the dots in pink colour represent QTNs detected by ≥2 models. Pl Ht, Plant height; DTF, Days to flowering; PYD, Plot yield

GAPIT (Genome Association and Prediction Integrated) tool [62] and R package mrMLM [63] were used to perform GWAS in 198 rice accessions to identify the candidate QTNs including MLM [64], CMLM [47], mrMLM [49], FASTmrMLM [52] and FASTmrEMMA [50] and confirm the true associations with rice yield. Kinship matrix utilized in the study was calculated using TASSELv5.2.82 software. In single-locus models, the critical threshold of significant association was set similar to recent GWAS studies [65–74]. In multi-locus GWAS, QTNs with Logarithm of Odds, LOD score of ≥ 3 were adopted as the significant SNPs associated with grain yield [8, 19, 75–77]. QTNs detected by both ML-GWAS models and SL-GWAS models were projected as candidates for rice yield related traits. Manhattan and QQ plots for all the three traits are presented in Fig. 5. Favourable allele of each of the commonly identified QTN was then identified using the QTN effect values and the genotype for code 1. In case, the QTN has a positive effect value, genotype for code 1 is considered the favourable SNP allele; in case the QTN has a negative effect, another genotype is considered favourable. Percentage of superior alleles was calculated for each of the common QTN. For every rice accession, proportion of favourable alleles in these QTNs was estimated as the count of favourable alleles divided by total count of QTNs and visualized as a heatmap.

### SNP annotations, prediction and enrichment analysis of putative candidates

The online rice databases *viz.*, Genome Annotation database for rice (MSUv7.0, http://rice.uga.edu/); Information Commons for Rice (IC4R, http://ic4r.org/) and Gramene QTL (https://www.gramene.org/) were used to annotate the genes around the common QTNs discovered by different GWAS models. For all the identified gene loci, regions within LD decay distance were used to search for probable rice yield associated genes. With a view to further comprehend the molecular basis, we conducted gene-set enrichment analysis using ShinyGOv0.77 graphical tool wherein their biological functions, localization within plant cells and signalling pathways are disclosed [78].

### Expression profile of candidate genes

The database BAR (The Bio-Analytical Resource for Plant Biology, https://bar.utoronto.ca/) was adopted to investigate all the candidates in different tissues to further illustrate the associations between genes and phenotypic differences. Subsequently, genes showing high expression in particular tissues were identified. Their homologs were also searched using the *Arabidopsis* Information Resource (TAIR) database (https://www.Arabidopsis.org/) and hypothesized on their possible functions using QuickGO tool (https://www.ebi.ac.uk/QuickGO/). The *Arabiodopsis* homologs expression analysis was then performed using the Affymetrix ATH1 whole genome genechip arrays normalized using the FPKM and Robust Multi-array average (RMA) method [79] at *Arabidopsis* eFP browser tool (https://bar.utoronto.ca/efp/cgi-bin/efpWeb.cgi). The python package bioinfokit was used to create a heatmap illustrating the FPKM values of the homologs identified.

### Analysis of haplotypes and phenotypic differences

To verify the associated locus between candidate genes and rice yield traits, SNP Seek software (https://snp-seek.irri.org/) was used to perform haplotype analysis considering the non-synonymous coding SNPs, then student’s t test was performed to test the significant variations among the haplotypes. The haplotypes revealed and phenotypic distribution of each yield trait were then depicted as boxplots using ggplot2 package in R Studio.

## Results

### Phenotypic variance and correlations among yield traits

Three grain yield related traits, PlHt, DTF, and PYD were measured to study the variation in the subset panel of 198 selected accessions of rice. The scatter plot generated revealed that rice accessions show large variation for all the studied yield traits (Fig. 3). The mean value values recorded for PlHt, DTF and PYD were 127.25cm, 98.38 cm, and 2333.19 kg/ha, correspondingly. Furthermore, PlHt showed a wider variation in comparison to DTF ranging from 72.10 to 187.90 cms. Measures of variance for all these traits derived from descriptive analysis are presented in Table S2. Meanwhile, the PYD with maximum CV at 56% indicated that PYD had the highest extent of variation. Correlations among the three yield related traits were observed. PlHt and DTF were positively correlated with each other and negatively but significantly correlated with PYD. The Pearson coefficients, in overall were ranging from low to moderate.

### Genotyping of selected subset of 3K panel

Genotypic data for 198 accessions of rice was retrieved from the 1M GWAS SNP dataset available at the Rice SNP Seek Database (http://snp-seek.irri.org/_download.zul). Followed by imputation in Beaglev5.4 software, 5,53,229 SNPs were filtered with a read depth of 10 and minor allele frequency >5% and mapped on the 12 rice chromosomes. Maximum SNPs were mapped on chromosome 1(66716) and minimum SNPs were mapped on chromosome 10 (30527) (Fig. 3a). SNP distribution of different loci was moderately uniform across the rice genome indicating suitability of filtered SNPs for molecular dissection of traits governing in rice (Fig. 3b).

### Genetic divergence, population structure and LD

Genetic diversity analysis was conducted to to infer the extent of genetic variablity available in selected subset of rice accessions. The summary of the results of diversity statistics presented in Table S3 point towards considerable variability in the 198 accessions selected for our GWAS study. The ThetaPerBP values ranged from 0.09 to 0.38 (maximum value for the SNP marker) with a mean value of 0.23. The PiperBP for all genetic loci in the rice subset ranged from 0.05 to 0.5, with an average value of 0.28551. The average minor allele frequency, observed heterozygosity, and major allele frequency considering all the 198 accessions were depicted. The Bayesian model-based pattern of population structure was defined using high quality 5,53,229 SNPs in STRUCTURE software. Structure runs from K = 3 to K = 7 using 5,53,229 SNP markers are shown in Figure S3. Applying Evanno et al. (2005) method, maximum ∆K/K value of 2 was selected (Fig. 4b) suggesting existence of two subpopulations in the selected subset of re-sequenced 3000 rice genomes (Fig. 4a). The two subpopulations identified comprised of 173 (87.37%) and 25 (12.63%) accessions, correspondingly. The larger cluster, cluster 1 consisting of *indica* rice accessions belonged to *indica* subpopulations while the smaller and diverse cluster 2 included accessions from *japonica, aus/boro* and *intermediate* type subpopulations. In the case of K = 3, rice accessions were grouped into three populations in conformity with NJ clustering, of which each population had all admixed individuals (Figure S3). Above K = 3, improving model fit with incremental K values, propose that these values fail to display any significant phylogenetic structure (Falush et al., 2003, Prichard et al., 2007). To explore the genetic differentiation due to population structure among the 198 rice accessions as reflected by these 5,53,229 filtered genome-wide SNPs, we employed Neighbor Joining (NJ) analysis. The clustering pattern of NJ analysis corroborated with the results of population structure of all the selected accessions though the accessions into three major subgroups (Fig. 4d). A total of 26.26% of rice accessions belonging to *ind1a, ind1b, ind2* and *ind3* were included in cluster 2. Whereas, cluster 1 was identified as the smallest cluster consisting of only 4.04% of *indica* rice accessions. It was predominated in accessions belonging to *indx* and *ind1b* subpopulations. However, cluster 3 was the largest cluster that constituted 69.69% of the total accessions. It was recognized as the most diverse with smaller statured to medium height accessions belonging to *indica, japonica, aus/boro* and *intermediate* type subpopulations. According to principal component analysis, there were three subpopulations in the selected subset of rice accessions as represented by population structure analysis (Fig. 4c)

The genome-wide LD decay for filtered SNPs was plotted as a scatterplot between the average r^2^ values against the physical map distance in mega basepairs. As shown in graphical display (Fig. S1), r^2^ value decreased with the increase in distance resulting in fastest decay within 100 kb, and then progressively slowed down, therefore we searched genomic ranges of common QTNs (50kb on either side of the QTN identified).

### QTNs identified for targeted yield traits using GWAS models

**Fig. 6:**
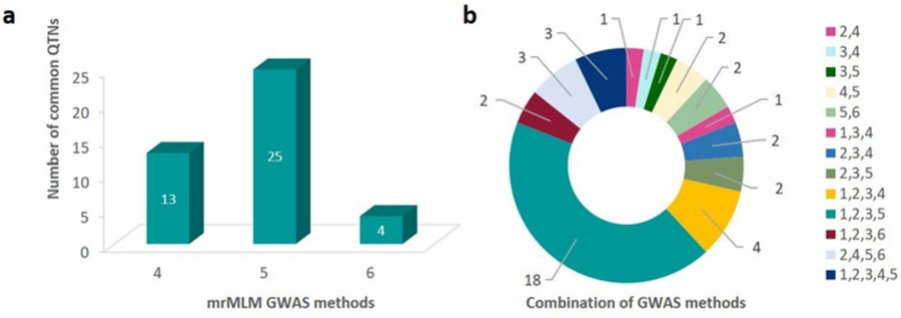
Total number of QTNs identified in the GWAS study. **a** QTNs identified simultaneously across the mrMLM methods; **b** QTNs identified by different combinations of GWAS methods. GWAS methods: 1, MLM; 2, CMLM; 3, MLMM; 4, mrMLM; 5, FASTmrMLM; 6, FASTmrEMMA

We analyzed all the three yield traits by employing six GWAS models to identify a total of 73 QTNs. Of these, 23,25 and 25 QTNs were associated with PlHt, DTF and PYD correspondingly. Manhattan plots portraying significant QTNs and the respective QQ plots for the abovementioned traits are shown (Fig. 5). Additionally, we identified the common QTNs across GWAS methodologies that were considered robust QTNs strongly associated with the three targeted yield traits. Accordingly, 15 common QTNs were detected concurrently by both SL- and ML approaches located on chromosome no 1,2,3,7,8,9,10,11 and 12 (Table 1). Their LOD scores ranged from 3.11 to 7.93 with consistent QTN effect values across different GWAS methods. Amongst the mrMLM GWAS methodologies, maximum number of common QTNs were identified by FASTmrMLM model (25) (Fig. 6a) and amongst the different combinations of GWAS models, MLM, CMLM, MLMM and FASTmrMLM identified the maximum number of QTNs (18) (Fig. 6b). We mapped one robust QTN on chromosome which was detected by all the three mrMLM GWAS models, 238555432 with variable LOD score values of 3.19 to 7.29 and percentage of variance explained (r^2^) ranging from 3.03 to 7.34 (Table 1).

**Table 1:** Common QTNs for three grain quantity traits in rice across different multi-methods

**Trait**	**Method**^**a**^	**Marker**	**Chr**	**Position (bp)**	**QTN effect**	**LOD score**	**r**^**2**^** (%)**^**b**^
Pl Ht	1,2,3,4	**41184096**	**1**	**41184096**	**7.82**	**5.76**	**5.64**
Pl Ht	5,6	**53308499**	**2**	**10037576**	**-3.06, -8.86**	**3.56,3.15**	**1.45,2.92**
PYD	1,2,3,5	**65474785**	**2**	**22203862**	**284.86**	**4.39**	**2.47**
PYD	1,2,3,5	77317239	2	34046316	257.91	3.91	2.24
PYD	1,2,3,5	**83271479**	**3**	**4063306**	**169.78**	**3.80**	**1.52**
Pl Ht	2,4,5,6	**238555432**	**7**	**26223525**	**7.43, 6.39, 8.97**	**7.29, 7.03, 3.19**	**7.34,6.29,3.03**
DTF	3,4	245045948	8	3016420	3.85	6.93	4.94
PYD	1,2,3,4,5	**280074957**	**9**	**9602407**	**-544.06**	**5.95**	**7.81**
DTF	1,2,3,4	293545796	10	60526	-4.74	7.07	7.43
Pl Ht	1,2,3,4	341803503	11	25110946	5.81	3.96	4.44
Pl Ht	3,5	**325332817**	**11**	**8640260**	**-10.84**	**4.82**	**6.28**
PYD	1,2,3,5	339876697	11	23184140	239.78	3.11	1.50
DTF	4,5	**359450698**	**12**	**13737035**	**-5.86, -3.57**	**5.02**	**7.85,3.5**
DTF	1,2,3,5	**368582572**	**12**	**22868909**	**-4.22**	**7.93**	**6.78**
PYD	1,3,4	**357969622**	**12**	**12255959**	**-587.86**	**3.72**	**8.61**

*Pl Ht* Plant height; *DTF* Days to flowering, *PYD* Plot yield

^a^MLM, CMLM,MLMM,mrMLM,FASTmrMLM,FASTmrEMMA were indicated by , respectively

^b^r^2^(%), proportion of total phenotypic variation explained by each QTN. Bold text indicates the QTNs appeared to be in vicinity of genes or QTLs associated with Pl Ht, DTF and PYD

Based on gene annotations and prior studies, 10 out of 15 common QTNs have been confirmed to be located within or in proximity of the known yield related genes *viz*., 41184096, 53308499, 325332817, 359450698, 357969622, 368582572, 65474785, 83271479, 238555432, 280074957 and the remaining 5 QTNs were newly discovered with phenotypic variance of 1.5% to 7.43% (Table 1). These results established the experimental accuracy of our study on QTN detection and also a rapid way to select genotypes with superior allelic compositions for breeding high yielding rice.

### Superior allele analysis in selected rice 3K subset

**Fig. 7:**
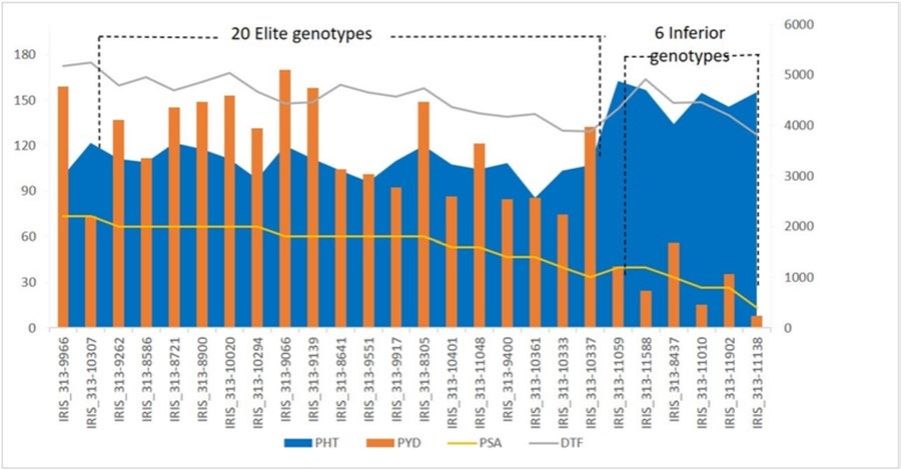
Distribution of elite alleles and yield traits in 26 higher and lower average phenotypic value genotypes

On the basis of average trait values of PlHt, DTF and PYD, we identified 20 elite genotypes having higher phenotypic values and 6 inferior genotypes with lower phenotypic values (Table 2, Fig. 7). The percentage of superior alleles across the 15 QTNs in each of the elite genotypes varied from 33.33% to 73.33% of which 80% (16 of 20 genotypes) displayed >50% PSA and the remaining 4 genotypes (20%) exhibited PSA <50%. On the other hand, the percentages for superior alleles in inferior genotypes fluctuated from 13.33% to 40% wherein all the 6 genotypes displayed PSA <50% indicating that 20 genotypes carry all the desired alleles for the targeted yield traits and can be employed in molecular breeding in rice.

**Table 2:** Phenotypic averages of plant height, days to flowering and plot yield and proportion of superior alleles in 26 rice genotypes across 15 common QTNs

**Line**	**PSA (%)**	**Pl Ht(cms)**	**DTF**	**PYD (kg/ha)**
IRIS_313-8305	60.00	120.10	97.75	4461.25
IRIS_313-8586	66.67	108.90	98.50	3352.50
IRIS_313-8641	60.00	103.60	100.12	3129.17
IRIS_313-8721	66.67	121.80	89.62	4363.33
IRIS_313-8900	66.67	117.50	95.50	4465.42
IRIS_313-9066	60.00	119.80	87.75	5098.33
IRIS_313-9139	60.00	111.50	88.75	4741.66
IRIS_313-9262	66.67	111.20	93.16	4105.42
IRIS_313-9400	46.67	108.70	92.50	2539.58
IRIS_313-9551	60.00	95.90	95.25	3033.33
IRIS_313-9917	60.00	109.70	92.25	2770.00
IRIS_313-9966	73.33	100.00	99.12	4777.08
IRIS_313-10020	66.67	111.40	101.50	4587.92
IRIS_313-10294	66.67	98.10	88.75	3949.58
IRIS_313-10307	73.33	121.90	101.50	2215.00
IRIS_313-10333	40.00	103.30	89.75	2242.92
IRIS_313-10337	33.33	107.00	96.25	3974.17
IRIS_313-10361	46.67	85.40	94.25	2563.75
IRIS_313-10401	53.33	107.60	92.00	2592.50
IRIS_313-11048	53.33	104.20	88.00	3645.42
**IRIS_313-8437**	**33.33**	**134.10**	**115.00**	**1680.83**
**IRIS_313-11010**	**26.67**	**154.90**	**122.00**	**453.75**
**IRIS_313-11059**	**40.00**	**162.91**	**104.50**	**1222.92**
**IRIS_313-11138**	**13.33**	**155.40**	**114.00**	**239.17**
**IRIS_313-11588**	**40.00**	**156.70**	**123.75**	**737.08**
**IRIS_313-11902**	**26.67**	**145.80**	**113.50**	**1061.67**

Bold font indicates the 6 lines with lower average values of Pl Ht, DTF, PYD and non-bold font indicates the 20 lines with higher average phenotypic values

*Pl Ht* Plant height; *DTF* Days to flowering, *PYD* Plot yield

**Fig. 8:**
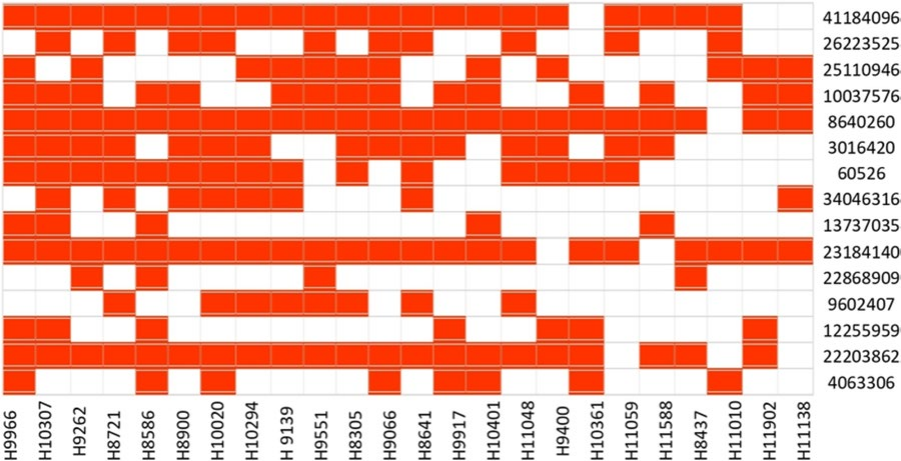
Heatmap presenting the distribution of superior allelic combinations for the 15 QTNs identified in 20-elite and 6-inferior genotypes. Red and white coloured blocks correspond to the superior and inferior alleles

Additionally, we discovered few common superior alleles in multiple elite genotypes. For instance, seven genotypes IRIS_313-9966, IRIS_313-9262, IRIS_313-9139, IRIS_313-9551, IRIS_313-8305, IRIS_313-9066, IRIS_313-8641 and IRIS_313-10401 carry all the superior alleles 41184096, 25110946, 100375576, 8640260, 23184140 and 22203862 and the superior alleles 8640260 and 22203862 occurred in all the 20 genotypes (Fig. 8). We predict that these common superior alleles might strongly influence the grain yield traits and generate valuable information for breeding improved rice in future.

### Excavation of candidate loci around robust QTNs identified

**Fig. 9:**
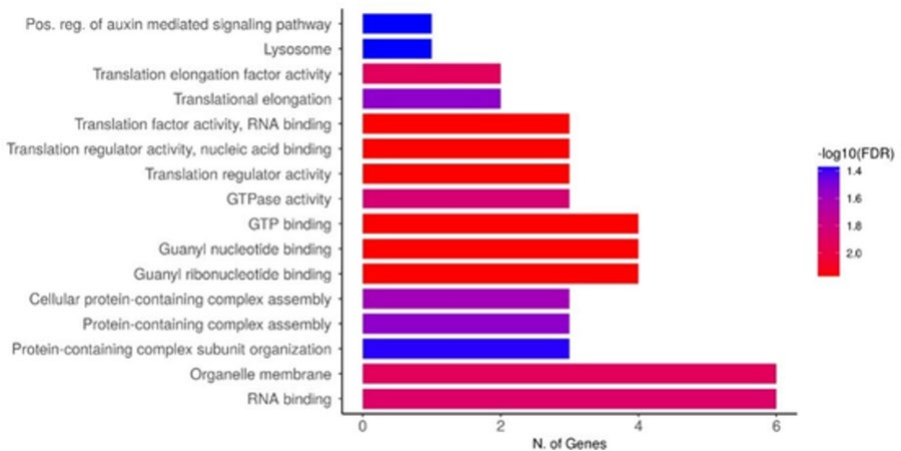
Barplot showing gene enrichment pathways for 73 candidate genes

We searched the probable CGs for each of the 15 QTNs. Consequently, we identified 239 genes and 73 genes were highly expressed in specific tissues *viz.,* shoot, leaves and rice seed grains and inflorescence as per the BAR tool (Table 3). Data on gene annotations revealed that 39 of 73 genes (53.42%) were annotated previously in 16 pathways using ShinyGOv0.77 tool (Fig. 9). Homologs of the 73 candidates were identified in model plant *Arabidopsis thaliana* using TAIR database and anticipated their putative roles (Table 4).

**Table 3:** Showing detailed information on pathway annotations of 73 candidate loci

**QTN name**	**Gene name**^**a**^	**Chromosome**	**Position**	**GO** **number**	**Annotation**
**41184096**	**LOC_Os01G71190**	**1**	**41186974..41188839**	**GO:0031090**	**Photosystem II reaction center PSB28 protein**
41184096	LOC_Os01G71106	1	41160726..41155813	˗	Disease resistance protein (TIR-NBS-LRR class) family
41184096	LOC_OS03G07960	3	4069589..4072442	GO:0031090	expressed protein
41184096	LOC_Os03G08020	3	4506887-4507439	GO:0003746	elongation factor Tu, putative, expressed
**10037576**	**LOC_Os02G17460**	**2**	**10049191..10042478**	**˗**	**tesmin/TSO1-like CXC domain containing protein, expressed**
**10037576**	**LOC_OS02G17520**	**2**	**10084266..10087922**	**GO:0034622**	**cytochrome c-type biogenesis protein ccmH precursor, putative, expressed**
10037576	LOC_OS02G17470	2	10050837..10057784	GO:0003723	RNA-binding protein-related, putative, expressed
22203862	LOC_OS02G36710	2	22174407..22181019	GO:0034622	galactosyltransferase family protein, putative, expressed
4063306	LOC_OS03G08050	3	4111409..4113361	GO:0006414	elongation factor Tu, putative, expressed
4063306	LOC_Os03G07994	3	4089784..4086997	˗	expressed protein
4063306	LOC_Os03G08000	3	4090003..4094134	˗	expressed protein
4063306	LOC_Os03G08010	3	4095194..4097239	˗	elongation factor Tu, putative, expressed
26223525	LOC_OS07G43890	7	26242366..26245311	GO:0031090	emp24/gp25L/p24 family protein, putative, expressed
**26223525**	**LOC_Os07G43870**	**7**	**26230679..26240915**	**˗**	**heat shock protein DnaJ, putative, expressed**
3016420	LOC_Os08G05670	8	3038465..3031282	˗	HEAT repeat family protein, putative, expressed
3016420	LOC_Os08G05530	8	2965358..2968015	˗	LSM domain containing protein, expressed
**3016420**	**LOC_Os08G05570**	**8**	**2977391..2982700**	**˗**	**monodehydroascorbate reductase, putative, expressed**
9602407	LOC_Os09G15790	9	9643529..9646172	GO:0003924	ras-related protein, putative, expressed
9602407	LOC_Os09G15775	9	9636409..9637761	˗	expressed protein
9602407	LOC_Os09G15760	9	9629854..9631896	˗	expressed protein
9602407	LOC_OS09G15770	9	9634451..9638121	˗	CPuORF13 - conserved peptide uORF-containing transcript, expressed
9602407	LOC_Os09G15720	9	9595103..9593570	˗	expressed protein
23184140	LOC_OS11G38959	11	23184447..23187634	GO:0003723	40S ribosomal protein S9-2, putative, expressed
23184140	LOC_Os11g38970	11	23189532..23190792	˗	expressed protein
23184140	LOC_Os11g38990	11	23212467..23217461	˗	peptidyl-prolyl cis-trans isomerase, putative, expressed
23184140	LOC_Os11g38880	11	23142279..23140918	˗	hypothetical protein
23184140	LOC_Os11g38910	11	23166977..23167787	˗	pyruvate decarboxylase isozyme 1, putative, expressed
23184140	LOC_Os11g38930	11	23176604..23178856	˗	tRNA-splicing endonuclease subunit Sen2, putative, expressed
60526	LOC_Os10g01030	10	40060..38648	˗	hypothetical protein
60526	LOC_Os10g01010	10	22576..22214	˗	transposon protein, putative, Ac/Ds sub-class, expressed
**60526**	**LOC_Os10g01110**	**10**	**103441..100327**	**˗**	**OsSCP44 - Putative Serine Carboxypeptidase homologue, expressed**
60526	LOC_Os10g01080	10	82250..80964	˗	SOR/SNZ family protein, putative, expressed
60526	LOC_Os10g01060	10	67119..72971	˗	protein kinase family protein, putative, expressed
60526	LOC_Os10g01044	10	58337..45941	˗	isoflavone reductase, putative, expressed
25110946	LOC_Os11g41820	11	25153456..25148703	GO:0003723	RNA recognition motif containing protein, expressed
8640260	LOC_Os11g15230	11	8598973..8596981	˗	expressed protein
8640260	LOC_Os11g15280	11	8635205..8631629	˗	TNP1, putative, expressed
**13737035**	**LOC_Os12g24170**	**12**	**13782378..13761534**	**˗**	**beta-galactosidase precursor, putative, expressed**
**13737035**	**LOC_Os12g24080**	**12**	**13697068..13702579**	**˗**	**HECT-domain domain containing protein, expressed**
13737035	LOC_Os12g24130	12	13735026..13738628	˗	expressed protein
22868909	LOC_Os12g37210	12	22827572..22824580	˗	expressed protein
12255959	LOC_Os12g21789	12	12267520..12264653	˗	expressed protein
**12255959**	**LOC_Os12g21710**	**12**	**12204428..12208058**	**˗**	**nnrU, putative, expressed**

Bold font signifies genes that we propose correlate with PlHt, DTF and PYD in rice

^a^Indicates the gene locus that correlates with the QTN (before the gene name in the same row)

**Table 4:** Details of 25 homologous genes in *Arabidopsis thaliana*

**Gene name**	**GO number**	**Homologous gene**	**Annotation**
**LOC_OS01G71190**	**GO:0031090**	**AT4G28660**	**Photosystem II reaction center PSB28 protein**
**LOC_OS02G55640**	**GO:0034622**	**AT1G67250**	**Proteasome maturation factor UMP1**
**LOC_OS02G17520**	**GO:0031090**	**AT1G15220**	**Cytochrome c biogenesis protein family**
**LOC_OS02G17470**	**GO:0003723**	**AT4G28990**	**RNA-binding protein-related**
**LOC_OS03G08020**	**GO:0003723**	**AT1G07920**	**GTP binding Elongation factor Tu family protein**
**LOC_OS03G08050**	**GO:0005525**	**AT1G07920**	**GTP binding Elongation factor Tu family protein**
**LOC_OS03G08000**	**˗**	**AT1G54150**	**E3 Ubiquitin ligase family protein**
LOC_OS03G08010	˗	AT1G07920	GTP binding Elongation factor Tu family protein
LOC_OS07G43890	GO:0031090	AT1G14010	Emp24/gp25L/p24 family/GOLD family protein
**LOC_OS07G43870**	**˗**	**AT1G18700**	**DNAJ heat shock N-terminal domain-containing protein**
**LOC_OS08G05670**	**˗**	**AT4G38120**	**ARM repeat superfamily protein**
**LOC_OS08G05570**	**˗**	**AT1G63940**	**Monodehydroascorbate reductase 6**
**LOC_OS09G15775**	**˗**	**AT1G36730**	**Translation initiation factor IF2/IF5**
**LOC_OS09G15770**	**GO:0005525**	**AT1G36730**	**Translation initiation factor IF2/IF5**
**LOC_OS11G41820**	**GO:0003723**	**AT1G60900**	**"U2 snRNP auxilliary factor, large subunit, splicing factor"**
**LOC_OS11G38959**	**GO:0003723**	**AT5G39850**	**Ribosomal protein S4**
**LOC_OS11G38990**	**˗**	**AT1G26940**	**Cyclophilin-like peptidyl-prolyl cis-trans isomerase family protein**
LOC_OS11G38930	˗	AT3G45590	Splicing endonuclease 1
**LOC_OS10G01110**	**˗**	**AT1G33540**	**Serine carboxypeptidase-like 18**
**LOC_OS10G01080**	**˗**	**AT2G38230**	**Pyridoxine biosynthesis 1.1**
LOC_OS10G01044	˗	AT3G18890	NAD(P)-binding Rossmann-fold superfamily protein
**LOC_OS12G24170**	**˗**	**AT2G32810**	**Beta galactosidase 9**
**LOC_OS12G24080**	**˗**	**AT1G55860**	**Ubiquitin-protein ligase 1**
**LOC_OS12G21789**	**˗**	**AT3G49880**	**Glycosyl hydrolase family protein 43**
**LOC_OS12G21710**	**˗**	**AT1G10830**	**15-cis-zeta-carotene isomerase**

Bold font shows genes that we suggest correlate with plant height, days to flowering and plot yield in rice

### Expression profile of the homologous genes

**Fig. 10:**
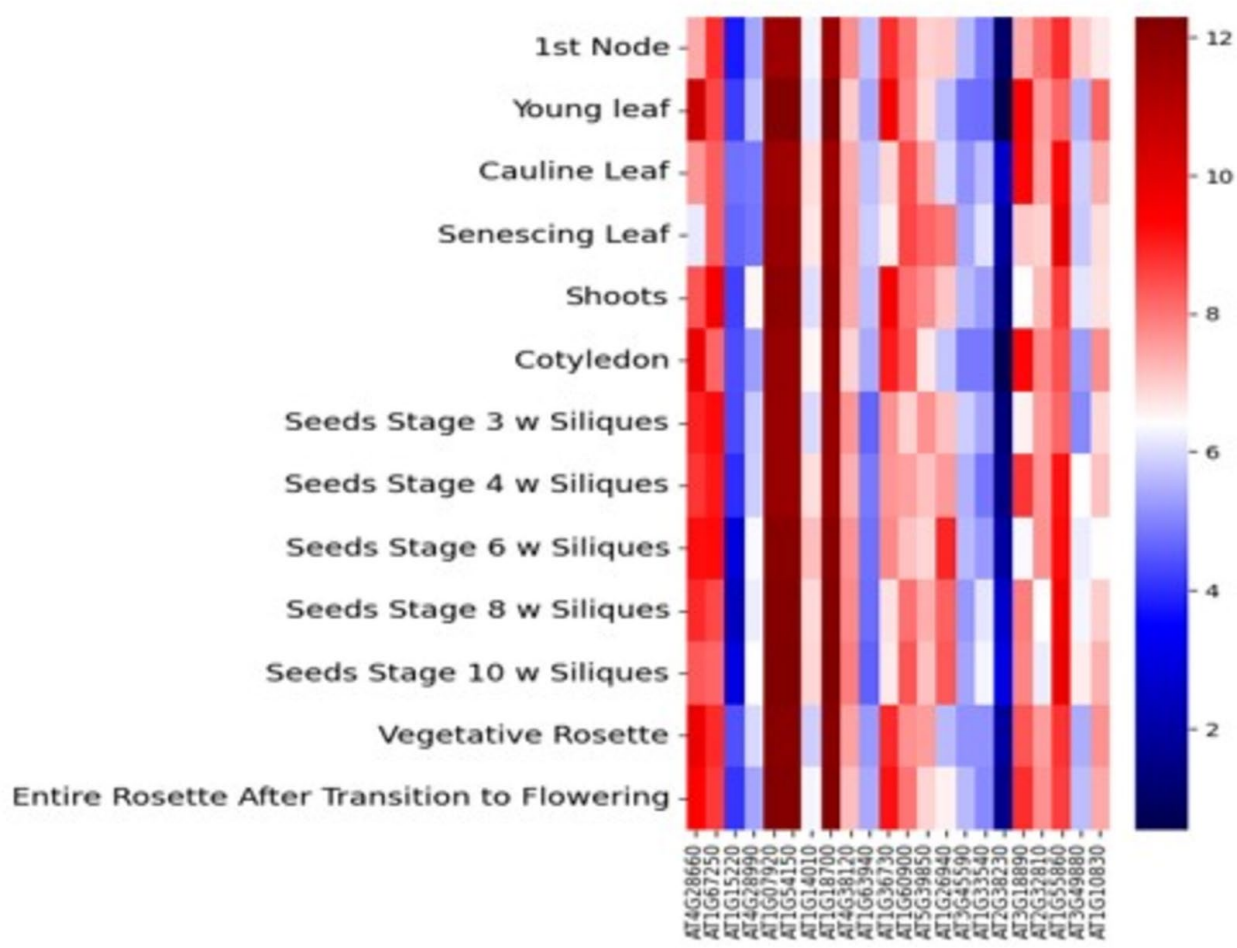
Heatmap depicting FPKM expression values of the 25 homologs identified for the three grain yield traits. The red color indicates high expression, and the blue color shows low expression. y-axis is log2 (FPKM +1)

The *Arabidopsis* eFP browser tool at BAR database demonstrates the normalized FPKM expression data obtained for specific tissues, including 1^st^ node, young leaves, mature leaf, shoots, cotyledons, seeds and rosette vegetative and after flowering. Heatmap of the homologous genes depicting the FPKM expression of the homologs in different tissues are shown in Fig. 10 (Table S4). As shown, AT1G07920 and AT01G18700 had the highest FPKM values in 1st node, young leaf, mature leaves, shoots, cotyledons, seeds and rosette. For flowering time QTNs, AT04G38120, AT01G63940, AT01G33540, AT02G38230, AT02G32810 and AT01G55860 had higher expression in vegetative rosette and rosette after flowering indicating a potential association between these candidates and flowering time. For PlHt QTNs, AT04G28660, AT01G67250, AT01G18700, AT01G60900, AT04G28990 and AT01G15220 had higher expression in cotyledons, seeds and rosette suggesting these genes may have specifically a large role on yield. Furthermore, AT01G07920, AT01G54150, AT01G36730, AT03G49880, AT05G39850, AT01G26940 and AT01G10830 found in QTNs for plot yield showed higher expressions in cotyledons, seeds and inflorescense, and were identified to associate with rice yield.

### Gene enrichment of identified candidates

**Fig. 11:**
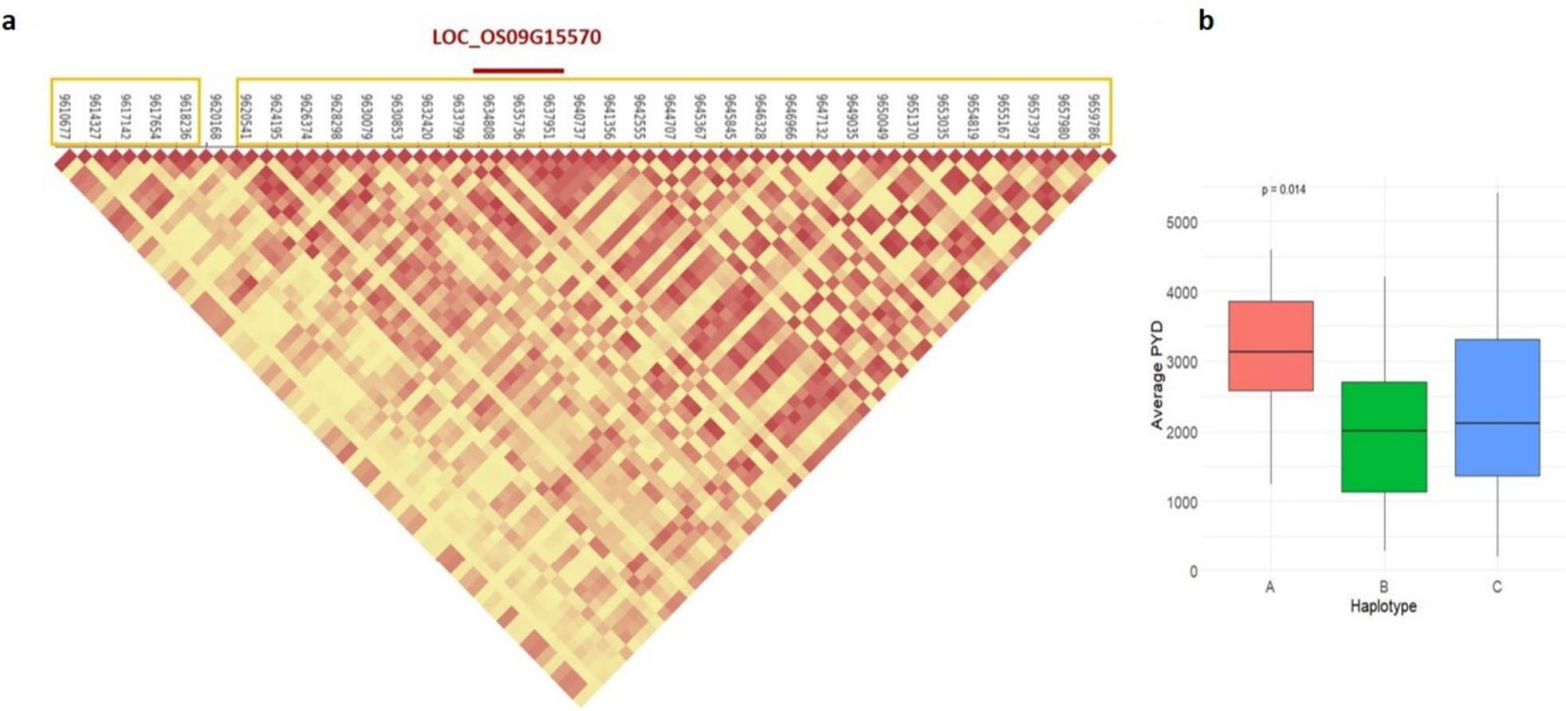
Gene ontology tree depicting annotations of putative SNP markers. Boxes in the diagram represent the GO terms corresponding to a seven-digit ID number preceded by GO and their functional description. Yellow coloured nodes at the bottom represent the significantly enriched GO terms in enrichment analysis. Arrowheads to the left and right indicate the direction of relationships between different GO terms/nodes at upper and lower levels

ShinyGO tool was used to conduct GO functional enrichment analysis of candidates underlying the targeted yield traits. According to enrichment analysis, 40 of 73 candidates were annotated and significantly enriched for 60 GO terms (*p* < 0.05). Out of 60 GO terms, 34,13 and 13 GO terms were enriched in the category of biological, cellular and metabolic component. The bar plot depicting the most significant pathways of these CGs are shown in red colour (Fig. 9). Genes enriched in biological processes comprised of GO terms associated with biogenesis, cell development, reproduction, anatomical structure development, regulation of metabolic processes, signalling and stress responses (Fig. 11). Interestingly, one GO term, GO:0010929, a positive regulator of auxin mediated signalling pathway also governs several other pathways mediating growth, reproduction and architecture of rice plants. The 25 genes involved in cellular processes included organelle membrane, protein complexes, envelope, photosynthetic and endomembrane system. Another enriched term, GO:0031090 was associated with different membrane-bound organelles within the cell. Among the molecular components, GO:0090079 with translation regulator and nucleic acids binding activity has a central role in initiation, activation and termination of polypeptide synthesis at the site of ribosomal assembly. These findings demonstrate the influence of these CGs on rice yield and provide valuable insight into their genetic basis and discover new yield related genes in rice.

### Haplotype and phenotypic difference analysis of candidate genes

**Fig.12:**
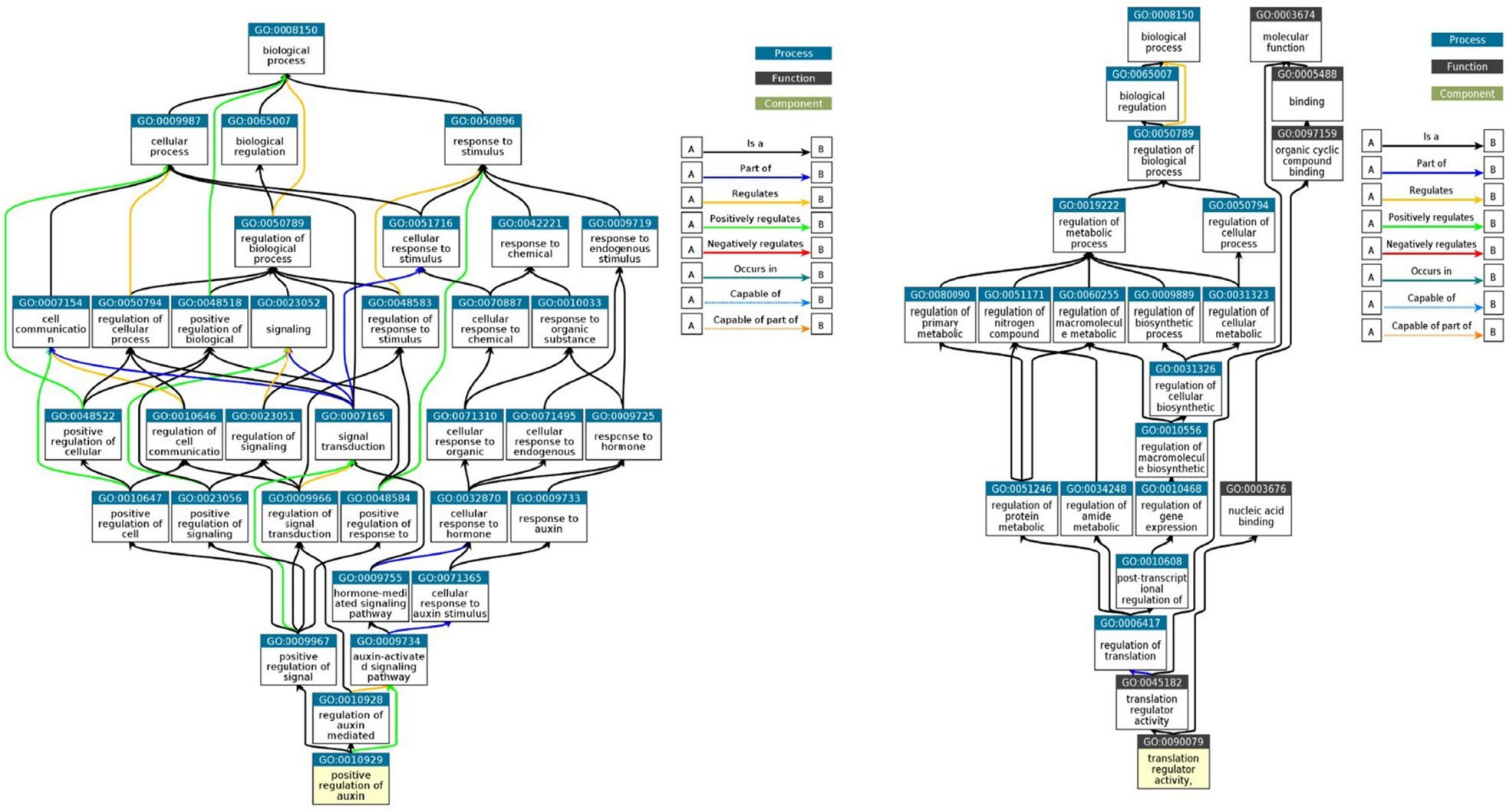
Results of haplotype and phenotypic difference analysis for the candidate gene LOC_OS09G15770. **a** LD and haplotype block with five SNPs within LOC_OS09G15770. **b** boxplot of PYD trait among the three haplotypes of LOC_OS09G15770. The SNP markers in LD region around the significant QTN (10037576) are shown in yellow color

To further confirm the association of the candidate loci and rice yield traits using SNPs within the candidate genes. LOC_OS09G15770 (9634451-9638121) was analyzed to reveal the intragenic variation affecting the yield in rice and to identify superior haplotype. Fig. 12(a) depicts the linkage disequilibrium and haplotype block with five SNPs at 9636961 bp, 9637028 bp, 9637406 bp, 9637426 bp and 9637435 bp. The 198 accessions were categorized into 3 haplotypes based on these 5 SNPs *viz.,* HapA (GTTCG), HapB (TCTCG), HapC (GCCCG). Amongst these haplotypes, HapA recorded the highest average PYD (3118.67) whereas the HapB presented the lowest average PYD (1932.77) (Fig. 12b). A student t test showed that significant differences existed between the haplotypes (*P*-value = 0.014). There was also a significant variation in PYD between haplotypes of LOC_OS02G36710 (*P*-value = 0.0017). Therefore, we deduce the candidate genes LOC_OS09G15770 and LOC_OS02G36710 to be associated with grain yield in rice. Supplementary Figure S2 depicts the results of haplotype block and phenotypic variation in LOC_OS02G17520, which was identified for plant height. We may presume that candidate loci LOC_OS09G15770, LOC_OS02G36710 and LOC_OS02G17520 might be associated with rice yield.

## Discussion

Improving the productivity has been a major bottleneck for rice breeders ensuring food supply to an ever-growing global population. Mostly, yield traits are complex and modulated by various polygenes. GWAS predictions on the gene loci governing rice yield traits based on different regression models and identification of associated SNP markers might reveal new insights into the molecular mechanism underlying rice yield. Classic models of GWAS have been employed widely to discover variations in a gene particularly in several crops [16, 80] although, such models suffer from critical test corrections and ignore the effect of multiple gene loci in overall [8, 81, 82] therefore, ML-models with higher statistical power were developed. In our investigation, we employed six different GWAS models including classic and ML-models both in a 3K-RGP subset of 198 accessions to detect QTNs associated with three grain yield traits (PlHt, DTF and PYD). Fifteen QTNs were identified by two or more GWAS models instantaneously (Table 1). Remarkably, we dissected multiple gene clusters for the targeted traits; for example, the SNP marker 65474785 on chromosomal number 3 at 22203862 bp, was located in close vicinity of genes controlling number of rice grains/plant, plant height and heading date, LOC_Os03g07920 (OsBG1) [83], LOC_Os03g07360 (dof12) [84], LOC_Os3g08850(AFB6) [85]. Moreover, 41184096 located on chromosome 1 showed overlap

 with LOC_Os01g71340 (OsGLU1) [86] encoding membrane bound endo-1,4-β-D-glucanase that participates in signalling pathways induced by plant hormones gibberellins (GA) and brassinosteroids (BR) and facilitates cell development. Glu1 mutants exhibit dwarfism due to defects in elongation of internodes in rice [5]. Furthermore, marker 245045948 at chromosome 8 was found to be closely associated with LOC_08g06110 (OsCCA1) regulating tiller production and panicle development in rice [7, 19]. These results demonstrated that the QTNs unravelled in our study possibly will be beneficial in modulating grain yield related traits in rice.

We studied pathway annotations and conducted GO analysis to identify the putative genes associated with rice yield (Table 3, text highlighted in bold). LOC_Os01G71190 (photosystem II reaction center protein, Psb28) belonging to the family of light induced genes in rice has regulatory role in stabilizing PSII activity. Knockouts of LOC_Os01G71190 caused a pale green phenotype and dwarfism [87]; might serve as a key gene for cell growth and development located near the OsGLU1 gene (157kb) that controls cell wall biosynthesis in coordination with GA and BR [5]. LOC_02G17460 is a member of polycomb group of proteins that encode TSO1-like cysteine rich (CXC) domain containing proteins highly expressed in the shoot apex, carpels, pollen, and seeds; works as a regulator of gene subsets involved in cell division and proliferation [88]. TSO1 insertional mutants also show phenotypic aberrations in pollen grains and carpels which indirectly affects the grain yield [89, 90]. LOC_Os02G17520 is a cytochrome c biogenesis ccmH precursor that has been confirmed as an essential housekeeping gene [91]. CCMH, a thiol-disulfide oxidoreductase within the mitochondrial membrane which is involved intricately in cytochrome c maturation and electron transfers between enzymes involved in photosynthesis and respiration [91]. Insertional mutation studies demonstrated lethal defects of knockout CCMH gene by arresting the embryo development at torpedo stage [92] and therefore, may play a crucial role in growth and embryo development. LOC_Os07G43870 codes for heat shock protein DnaJ that improves rice architecture by modulating gibberellin homeostasis [93] thereby, enhancing rice yield. Notably, a loss of function mutation (*NAL11*^−923del−1552^) in Narrow Leaf 11 gene, HSP with DnaJ domain results in GA defects and inhibits chloroplast development suggesting the role of LOC_Os07G43870 and gibberellins in determining the productivity in rice [94]. LOC_08G05570 is a monohydrate reductase gene (MDHAR) that participates in the ascorbate-glutathione cycle and mutants generated by RNAi exhibited decreased chlorophyll, relative ascorbate-dehydroascorbate ratios which in turn reduce biomass and rice yield [95]. LOC_Os10G01110 (OsSCP44) is annotated as a putative serine carboxypeptidase homologue that has a strong association with grain weight, therefore, likely to regulate rice yield. Serine peptidases with SP10 domain are thought to be involved in protein degradation and activate flowering genes by cleaving floral repressors [96]. LOC_Os10G01060 (a type of kinase family protein), LOC_Os02G55640 (proteasome maturation factor, UMP1), LOC_OsO3G08000(expressed protein) and LOC_Os03G08010 (elongation factor Tu) maintain a balance between growth, development and stress responses in plants by acting as sensors for different plant hormones and influencing grain yield components [97]. Recent studies revealed that plants with enhanced expression of receptor like-kinases (RLKs) displayed an increase in seed yield and induced earlier flowering [2, 55, 98–104]. LOC_Os12G21789 encodes a protein belonging to glycosyl hydrolase family that primarily works in strigolactone (SL) signalling pathway and is predicted to be closely related to rice yield traits. RNAi knockdown mutant OsD14L showed a reduction in size of panicles, seed grains leading to high tillering and dwarf phenotype in rice [3, 7, 105–107]. LOC_Os12G24170 is a precursor of beta-galactosidase enzyme (BGAL) that mediates the physiological process of seed germination through cross talks among GA and Abscisic acid (ABA) to determine grain yield in rice [3]. Knockout studies revealed that BGAL9 regulates different physiological functions and stress responses in rice [108]. Transcriptomics studies very well implied the upregulation of OsBAGL1, OsBAGL4, OsBAGL8 and OsBAGL11 genes during seed germination and their influence on plant growth and development [109]. LOC_Os12G24080 annotated as HECT-domain containing protein belonging to the family of Ubiquitin ligases (E3s) that play a crucial role in multiple biological processes which includes flowering [110, 111]. HECT ligases associated with proteasomes orchestrate plant growth and development severely [112]. HAF1, E3 ubiquitin ligase interacts with Heading date1(Hd1) gene and alters the flowering times in short- and long-day rice plants [113] signifying that HAF1 is critical to precisely modulate timing of Hd1 accumulation for the photoperiod induction. Another study revealed that Flowering Related RING Protein 1 (*FRRP1*), a E3 ubiquitin ligase possibly regulates flowering time and rice yield by reducing monoubiquitination of H2B histone proteins and resultant changes in length of grains, panicles and plant height [114]. LOC_Os12G21710 annotated as the high-tillering and dwarf 12 gene (htd12) encodes a 15-cis-carotene isomerase (Z-ISO) protein, a member of nitrite and nitric oxide reductase U (NnrU) family pf proteins that regulates carotenoids and SL biosynthetic pathways and modifies plant architecture [115]. T20, a carotene isomerase localized in chloroplasts suppresses outgrowth of rice tillers by cross talk among carotenoids, SL and ABA [116]. MIT3 gene coding for a carotenoid isomerase, mutants revealed high tillering, leaf variegations and a dwarfed phenotype implicating the role of carotene isomerases in governing rice yield by attuning the signalling pathways [11]. These putative genes appear to be closely associated to rice yield traits and could be utilized to develop functional markers for use in rice breeding programs.

We identified 25 *Arabidopsis* homologues to the 73 rice CGs, nineteen of which with higher expression in distinct tissues, such as leaves, shoots, cotyledons, seed stages, vegetative rosette and rosette after flowering were identified to affect grain yield in rice (Table 4). LOC_Os01G71190 seems to involve in GO:0031090. The homologous gene in *Arabidopsis* is AT4G28660, which governs different biological processes affecting the plant growth [117, 118]. LOC_Os02G55640 participates in the GO:0034622 pathway and its homologue in *Arabidopsis*, AT1G67250 have roles in protein degradation pathway (ERAD) and assembly of proteasomes with documented associations with grain yield [82, 119]. Ortholog of LOC_Os02G17520(Cytochrome c biogenesis family protein) with QTN 53308499 in its genic region is characterized by ABC-type transporter and ATP hydrolysis activity. Its involvement in ABA mediated pathways [120] and ATP binding, suggests potential association with rice development [46, 121]. Interestingly, there are two candidates, LOC_Os02G17470 (RNA binding protein-related) and LOC_Os07G43870(DNA J heat shock protein) gene which act as a positive regulator of pollen and pollen tube growth which is essentially important for reproduction in plants [122, 123]. Also, LOC_Os03G08000 (E3 ubiquitin ligase family protein), involved in proteolysis and regulation of developmental processes, has been found to be located near to the QTN, 83271479 associated with PYD and PlHt indicating a possible role in grain yield [9, 124, 125]. Interesting, yield related gene monohydrate reductase gene LOC_Os08G05770, was observed in neighbouring region of the QTN associated with DTF, signifying a possible pleotropic effect on all the grain yield related traits [38, 126–128]. Extensive studies revealed another CG, LOC_Os12G21710 encodes a 15-cis-zeta-carotene isomerase ortholog in *Arabidopsis* and it participates in unique functions in photosynthesis, signalling pathways via carotenoid derivatives and govern plant physiology, growth and stress responses in plants [1, 129, 130]. In *Arabidopsis*, serine-carboxypeptidase like-18 gene, LOC_Os10G01110, belonging to SCPL family is assumed to regulate seed germination and yield traits in rice *viz.,* grain weight and grain size due to SNP polymorphisms in allele *GS5-1* and *GS5-2* alleles in response to ABA [75, 131, 132]. Amongst the other notable yield related genes near the locus include LOC_Os12G24170 (BGAL9) and LOC_Os12G21789 (Glycosyl hydrolase family protein 43) which has a key role in plant cell growth and development, explicitly for microspore and pollen germination by modifying the cell wall components [133–135].

In the current study, six GWAS models were employed for yield related traits on selected subset of rice accessions using 5,53,229 SNPs and the average phenotypic data recorded in the IARI field. For plant height, 23 QTNs were identified, whereas 25 QTNs were identified for plot yield and days to flowering each. Amongst the 73 different QTNs in total, 24 were co-localized with already reported QTLs or loci in previous mapping studies. We obtained 15 significant QTNs and 10 candidates underlying the three aforementioned yield traits. Meanwhile, we selected 20 elite genotypes for breeding high yielding rice and identified 25 homologues of *Arabidopsis*, 19 of which might regulate yield traits in rice. Subsequently, gene annotation, gene ontology and enrichment analysis showed 40 CGs were found to be enriched in GO terms of the studied traits. LOC_OS09G15770, LOC_OS02G36710 and LOC_OS02G17520 were confirmed as key candidates by tissue-specific expression analysis, and haplotype and phenotypic variation analysis. Selecting the identified elite genotypes with increased frequency of desirable alleles for grain yield traits might speed up the rhythm of rice improvement and address the challenges relating to food security and sustainable rice production. Furthermore, the identification of QTNs and CGs in our study provided insights into the regulatory mechanisms and genetic associations of these yield related traits in rice.

### Supplementary Information

**Supplementary material 1.** 

**Supplementary material 2.** 

**Supplementary material 3.** 

**Supplementary material 4.** 

**Supplementary material 5.** 

**Supplementary material 6.** 

**Supplementary material 7.** 

## Data Availability

The genotype datasets analysed in our GWAS study are available at the Rice SNP-Seek Database (https://snp-seek.irri.org/) and the details of the genotypes used in the study can be found in supplementary information file.

## Abbreviations

GWAS: Genome wide-association study
SNPs: Single nucleotide polymorphisms
QTN: Quantitative trait nucleotide
SL: Single-locus
ML: Multi-locus
PlHt: Plant height
DTF: Days to 50% flowering
PYD: Plot yield
CV: Coefficient of variation
PiPerBP: Nucleotide diversity per base pair/ Genetic diversity
ThetaPerBP: The expected number of polymorphic sites per nucleotide
LD: Linkage Disequilibrium
H_o_: Observed heterozygosity
NJ: Neighbour joining
PCA: Principal component analysis
GAPIT: Genome Association and Prediction Integrated Tool
LOD: Logarithm of Odds
MSUv7.0: Genome Annotation database for Rice
IC4R: Information Commons for Rice
BAR: Bio-Analytical Resource for Plant Biology
RMA: Robust Multi-array average
TAIR: *Arabidopsis* Information Resource
GA: Gibberellins
BR: Brassinosteroids
SL: Strigolactone
BA: Abscisic acid
CG: Candidate gene
GO: Gene ontology
