# Supplementary figures and images for: New insights into QTNs and potential candidate genes governing rice yield via a multi-model genome-wide association study
Source: BMC Plant Biol. 2024 Feb 20;24:124. doi: 10.1186/s12870-024-04810-5 (PMC10877931; doi:10.1186/s12870-024-04810-5)

**Figure S1.** LD decay plot of subset of 3K-RGP, the average R2 plotted against distance in mega bases.


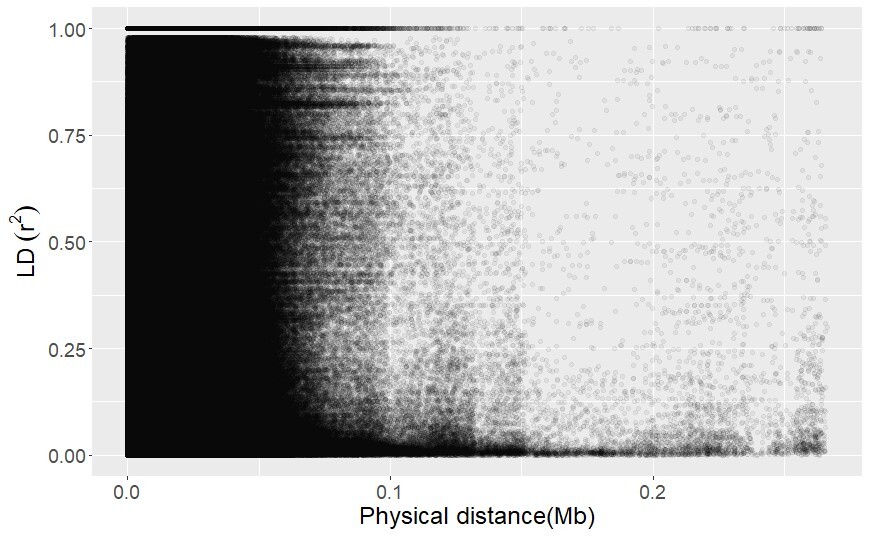

Supplement: Supplementary file 1 — Supplementary material 1. [file 12870_2024_4810_MOESM1_ESM.docx]
