# Supplementary material for: New insights into QTNs and potential candidate genes governing rice yield via a multi-model genome-wide association study
Source: BMC Plant Biol. 2024 Feb 20;24:124. doi: 10.1186/s12870-024-04810-5 (PMC10877931; doi:10.1186/s12870-024-04810-5)

**Figure S2.** Results of haplotype and phenotypic difference analysis for the candidate gene LOC_OS02G17520. (a) LD and haplotype block with one SNP within LOC_OS02G17520. (b) boxplot of PlHt trait among the three haplotypes of LOC_OS02G17520. The SNP markers in LD region around the significant QTN(10037576) are shown in yellow color.


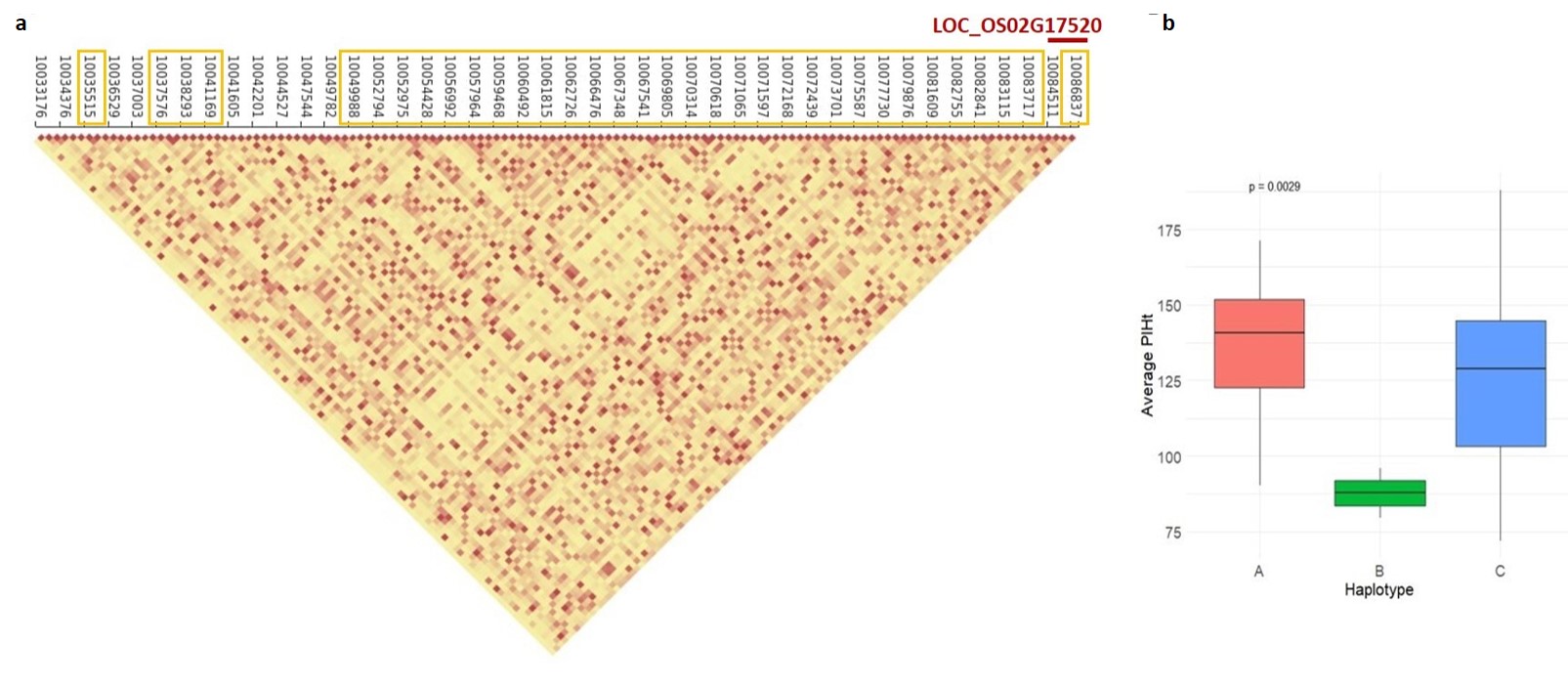

Supplement: Supplementary file 2 — Supplementary material 2. [file 12870_2024_4810_MOESM2_ESM.docx]
