# Supplementary material for: New insights into QTNs and potential candidate genes governing rice yield via a multi-model genome-wide association study
Source: BMC Plant Biol. 2024 Feb 20;24:124. doi: 10.1186/s12870-024-04810-5 (PMC10877931; doi:10.1186/s12870-024-04810-5)

**Figure S3.** Structure bar plots for K = 3-7. Each plot was created from 198 rice genotypes; each single vertical line represents each genotype, and each color represents one cluster.


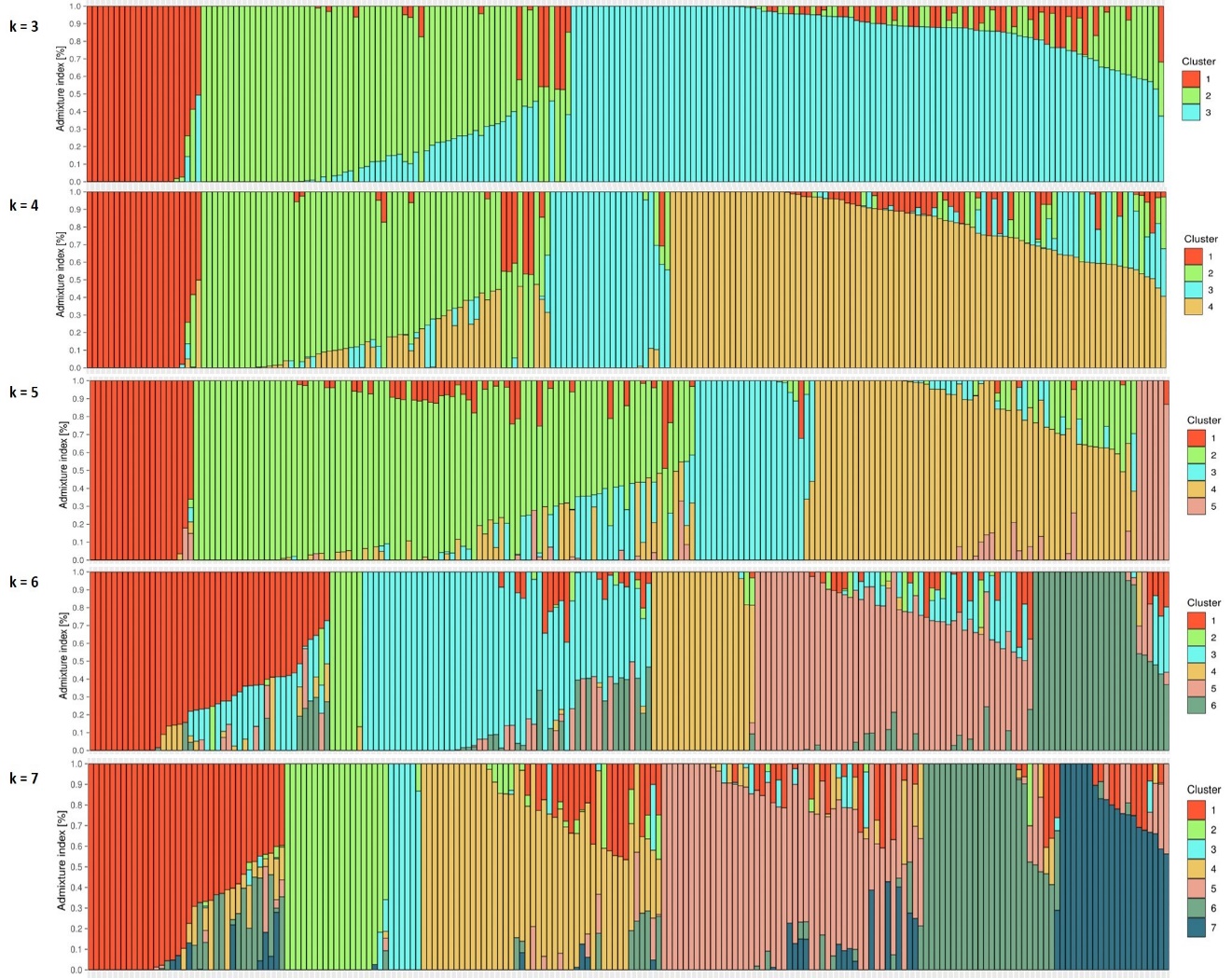

Supplement: Supplementary file 3 — Supplementary material 3. [file 12870_2024_4810_MOESM3_ESM.docx]
