# Supplementary material for: New insights into QTNs and potential candidate genes governing rice yield via a multi-model genome-wide association study
Source: BMC Plant Biol. 2024 Feb 20;24:124. doi: 10.1186/s12870-024-04810-5 (PMC10877931; doi:10.1186/s12870-024-04810-5)
Supplement: Supplementary file 4 — Supplementary material 4. [file 12870_2024_4810_MOESM4_ESM.docx]

**Table S1:** Details of 196 rice accessions used in the present study.

| **Name** | **Subpopulation*** | **Genetic stock_vername** | **Country source** |
| --- | --- | --- | --- |
| IRIS 313-9557 | ind2 | MULLIKURUVA::IRGC 77529-1-1 | India |
| IRIS 313-10403 | indx | IRGA 659-1-2-2-2::IRGC 117345-1-1 | Colombia |
| IRIS 313-9218 | ind2 | KALO CHAKOL::IRGC 77258-1-1 | Bangladesh |
| IRIS 313-9778 | admix | GENIT::IRGC 3272-1-1 | Argentina |
| IRIS 313-10170 | ind1A | MIN ZAO 6::IRGC 63772-1-1 | China |
| IRIS 313-11957 | indx | DAW LUEY 4-3::IRGC 76929-1 | Thailand |
| IRIS 313-10352 | indx | CT 9737-6-1-1-2-2P-M::IRGC 117330-1-1 | Colombia |
| IRIS 313-10148 | indx | CAUVERY::IRGC 45255-1-1 | India |
| IRIS 313-9019 | ind3 | KAM PAI::IRGC 78245-1-1 | Thailand |
| IRIS 313-10226 | indx | TONG GU HONG::IRGC 81026-1-1 | China |
| IRIS 313-9072 | ind2 | MUTA GANJE::IRGC 26744-1-1 | Bangladesh |
| IRIS 313-10179 | indx | KAMRANGA::IRGC 26373-2 | China |
| IRIS 313-7736 | ind2 | NONA BOKRA::IRGC 22710-C1-G1 | India |
| IRIS 313-8721 | aus | HOLOI BASH (SOLOI BASH)::IRGC 64778-1-1 | Bangladesh |
| IRIS 313-10361 | indx | IR 63295-AC 209-7::IRGC 117365-1-1 | Philippines |
| IRIS 313-9422 | aus | CHUNGUR BALI::IRGC 25855-1-1 | Bangladesh |
| IRIS 313-10334 | ind1B | B 6136 E 3-TB-0-1-5::IRGC 117311-1-1 | Indonesia |
| IRIS 313-10809 | trop | GAO JIAO HONG::IRGC 80920-1 | Indonesia |
| IRIS 313-11905 | ind3 | QINOGNAS::IRGC 8135-1 | Indonesia |
| IRIS 313-10341 | indx | BR 5230-46-4::IRGC 117318-1-1 | Bangladesh |
| IRIS 313-9384 | ind2 | BARIK KUDI::IRGC 52807-1-1 | India |
| IRIS 313-7780 | indx | SONA::IRGC 26971-C1-G1 | India |
| IRIS 313-11431 | ind3 | C 166-135::IRGC 50633-1 | Philippines |
| IRIS 313-11078 | indx | NON HAI::IRGC 29636-1 | Laos |
| IRIS 313-11902 | ind3 | CEMPO TURI::IRGC 73667-1 | Indonesia |
| IRIS 313-11242 | ind2 | OR 117-8::IRGC 39680-1 | India |
| IRIS 313-9925 | ind1B | MILYANG 30::IRGC 46977-1-1 | South Korea |
| IRIS 313-9966 | ind1B | CICA 9::IRGC 53079-1-1 | Colombia |
| IRIS 313-8341 | indx | BAT DO::IRGC 7014-1-1 | Vietnam |
| IRIS 313-9730 | ind1A | BA SHI ZAO::IRGC 67903-1-1 | China |
| IRIS 313-8660 | ind2 | KOTTEYARAN::IRGC 47383-1-1 | Sri Lanka |
| IRIS 313-11384 | indx | CHIEM TONG NHAT 1::IRGC 47496-1 | Vietnam |
| IRIS 313-9572 | indx | ASU::IRGC 62154-1-1 | Bhutan |
| IRIS 313-10400 | ind1B | IRGA 370-38-1-1F-C4-2::IRGC 117342-1-1 | Colombia |
| IRIS 313-10899 | ind3 | DAMNOEUB KHSE SAUT::IRGC 22819-1 | Cambodia |
| IRIS 313-10300 | indx | TANALA::IRGC 79467-1 | Colombia |
| IRIS 313-10002 | ind1B | BW 295-5::IRGC 63098-1-1 | Sri Lanka |
| IRIS 313-10040 | ind1B | MILYANG 77::IRGC 69340-1-1 | South Korea |
| IRIS 313-11056 | aus | AUS 301::IRGC 29089-1 | Bangladesh |
| IRIS 313-9822 | ind1A | CRILLO LA FRIA::IRGC 10793-1-1 | Venezuela |
| IRIS 313-8414 | indx | ARC 18202::IRGC 42328-1-1 | India |
| IRIS 313-9629 | aro | JC 157::IRGC 9074-1-1 | India |
| IRIS 313-8940 | ind1A | FU ZAO XIAN::IRGC 63619-1-1 | China |
| IRIS 313-10811 | indx | SEMPOR::IRGC 18761-1 | Indonesia |
| IRIS 313-8679 | ind3 | JAO LEUANG::IRGC 65866-1-1 | Thailand |
| IRIS 313-9201 | trop | ARC 6044::IRGC 12190-1-1 | India |
| IRIS 313-10046 | ind1B | CHAMA (DWARF)::IRGC 69487-1-1 | Zambia |
| IRIS 313-10177 | indx | DA GANG ZHAN::IRGC 67103-1-1 | China |
| IRIS 313-8454 | ind1A | LAI YIP ZIM::IRGC 4955-1-1 | Taiwan |
| IRIS 313-8932 | ind3 | PATISAIL::IRGC 37562-1-1 | Bangladesh |
| IRIS 313-8924 | ind2 | KUTTA::IRGC 52184-1-1 | India |
| IRIS 313-11423 | indx | C 1016-1::IRGC 50368-1 | Philippines |
| IRIS 313-11059 | aus | AUS 344::IRGC 29131-1 | Bangladesh |
| IRIS 313-10167 | indx | IR 13429-109-2-2-1::IRGC 63491-1-1 | Philippines |
| IRIS 313-9137 | aus | ARC 10100::IRGC 20709-1-1 | India |
| IRIS 313-9706 | ind1A | TAIPEI WOO CO::IRGC 112-1-1 | Taiwan |
| IRIS 313-12246 | ind3 | BANGKOUY::IRGC 94037-1 | Cambodia |
| IRIS 313-10620 | trop | BOW SU SO::IRGC 78237-1 | Brazil |
| IRIS 313-9522 | indx | RPW 9-4 (SS 1)::IRGC 50690-1-1 | India |
| IRIS 313-9368 | aus | CHANDARHAT::IRGC 25845-1-1 | Bangladesh |
| IRIS 313-11048 | aus | AUS 171::IRGC 29004-1 | Bangladesh |
| IRIS 313-10271 | indx | IA CUBA 17::IRGC 116990-1-1 | Cuba |
| IRIS 313-10996 | indx | BAKASI::IRGC 27074-1 | Indonesia |
| IRIS 313-10026 | ind2 | KITRANA 1007::IRGC 68517-1-1 | Madagascar |
| IRIS 313-12039 | ind3 | ITA 131::IRGC 80025-1 | Cambodia |
| IRIS 313-8412 | indx | FEI GAI 122::IRGC 63599-1-1 | China |
| IRIS 313-9758 | ind1A | I KUNG PAO::IRGC 114-1-1 | Taiwan |
| IRIS 313-8586 | ind3 | PLI KHAO::IRGC 64596-1-1 | Thailand |
| IRIS 313-10171 | ind1A | YA NONG ZAO 4::IRGC 63908-1-1 | China |
| IRIS 313-9935 | ind2 | MEKENZIE SMALL::IRGC 49895-1-1 | Guyana |
| IRIS 313-10940 | ind3 | J 6 IR 520 (WC 693)::IRGC 57600-1 | Indonesia |
| IRIS 313-8812 | ind3 | RELLY::IRGC 14623-1-1 | Indonesia |
| IRIS 313-11615 | ind3 | MANARE (PHOM)::IRGC 61437-1 | Guinea |
| IRIS 313-8305 | ind2 | URAIBOOL::IRGC 52785-1-1 | India |
| IRIS 313-10401 | ind1B | IRGA 370-42-1-1F-C-1::IRGC 117343-1-1 | Colombia |
| IRIS 313-8731 | ind2 | NIBARI::IRGC 67742-1-1 | India |
| IRIS 313-8530 | ind2 | DHANE BURWA::IRGC 10105-1-1 | India |
| IRIS 313-9391 | ind2 | KALABAIL::IRGC 25877-1-1 | Bangladesh |
| IRIS 313-10129 | ind1A | SAN SHIH TSI::IRGC 1038-1-1 | China |
| IRIS 313-11517 | ind1B | IR 4432-28-5::IRGC 55952-1 | Philippines |
| IRIS 313-10151 | ind3 | RD 15::IRGC 47705-1-1 | Thailand |
| IRIS 313-9259 | ind2 | G 25::IRGC 45733-1-1 | India |
| IRIS 313-9032 | ind1B | KHAO GRADOOK CHAHNG::IRGC 17111-1-1 | Thailand |
| IRIS 313-11054 | aus | AUS 295::IRGC 29083-1 | Bangladesh |
| IRIS 313-10257 | indx | ALTAMIRA 9::IRGC 116953-1-1 | Nicaragua |
| IRIS 313-10983 | indx | BOROJYOT::IRGC 26578-1 | Bangladesh |
| IRIS 313-9469 | ind1A | TSAO SHENG LI 1::IRGC 1309-1-1 | China |
| IRIS 313-8641 | aus | AUS 439::IRGC 29221-1-1 | Bangladesh |
| IRIS 313-8900 | ind2 | ARC 14654::IRGC 41663-1-1 | India |
| IRIS 313-11588 | ind3 | MR 69::IRGC 60188-1 | Malaysia |
| IRIS 313-10279 | indx | IR 3839-1::IRGC 55946-1 | Dominican Republic |
| IRIS 313-9732 | ind2 | MAMORIAKA::IRGC 68672-1-1 | Madagascar |
| IRIS 313-12040 | ind3 | NEANG LAU::IRGC 81328-1 | Cambodia |
| IRIS 313-8914 | indx | E 2040::IRGC 67968-1-1 | China |
| IRIS 313-10736 | aus | RERM BILASH::IRGC 16273-1 | Nepal |
| IRIS 313-8963 | aus | BATHURI::IRGC 25838-1-1 | Bangladesh |
| IRIS 313-9066 | ind1B | KULA KARUPPAN::IRGC 55328-1 | Bangladesh |
| IRIS 313-10333 | ind1B | B 6136-3-TB-0-1-5::IRGC 117312-1-1 | Indonesia |
| IRIS 313-10221 | indx | BA BAI GU::IRGC 79580-1-1 | China |
| IRIS 313-9634 | indx | ARC 15873::IRGC 43250-1-1 | India |
| IRIS 313-8968 | ind2 | KALU ILANKALAYAN::IRGC 36270-1-1 | Sri Lanka |
| IRIS 313-10189 | ind1A | DA NUO (ZHAN)::IRGC 72025-1-1 | China |
| IRIS 313-9626 | aus | KALIA::IRGC 34699-1-1 | Bangladesh |
| IRIS 313-10287 | ind1B | UQUIHUA::IRGC 117037-1-1 | Peru |
| IRIS 313-9429 | indx | RTS 16::IRGC 8235-1-1 | Vietnam |
| IRIS 313-11515 | indx | BKN BR 1031-78-5-4::IRGC 55927-1 |  |
| IRIS 313-8846 | indx | BAIANG 6::IRGC 6129-1-1 | Indonesia |
| IRIS 313-8699 | indx | BALASURIYA A::IRGC 66509-1-1 | Sri Lanka |
| IRIS 313-10237 | ind1B | PSBRC 86::IRGC 99716-1-1 | Philippines |
| IRIS 313-8994 | indx | ARC 14064::IRGC 41377-1-1 | India |
| IRIS 313-8930 | ind1B | MUKKALA BAZAL::IRGC 77279-1-1 | Bangladesh |
| IRIS 313-9403 | ind2 | BADUIE::IRGC 53715-1-1 | India |
| IRIS 313-8405 | indx | JIN JUN DAO::IRGC 59710-1-1 | China |
| IRIS 313-9551 | ind2 | BENGALY MORIMO::IRGC 10976-1-1 | Madagascar |
| IRIS 313-10294 | indx | IR 21015-72-3-3-3-1::IRGC 117004-1-1 | Philippines |
| IRIS 313-8793 | ind3 | KHAO THI RATE::IRGC 58041-1-1 | Myanmar |
| IRIS 313-10842 | ind3 | LUBUK LINGGAU::IRGC 20000-1 | Indonesia |
| IRIS 313-9611 | ind2 | WANGA BARUGULU::IRGC 52261-1-1 | India |
| IRIS 313-11692 | ind1A | CHIAYI WU-K'O::IRGC 64974-1 | Taiwan |
| IRIS 313-9924 | indx | KN 1 B 361-1-8-6-9::IRGC 46974-1-1 | South Korea |
| IRIS 313-10020 | aus | HODARAWALA::IRGC 67631-1-1 | Sri Lanka |
| IRIS 313-8603 | indx | ARC 12884::IRGC 22417-1-1 | India |
| IRIS 313-10274 | ind1B | ICTA CRISPO 38::IRGC 116994-1-1 | Guatemala |
| IRIS 313-8437 | indx | IRRIBINI::IRGC 49094-1-1 | Bangladesh |
| IRIS 313-10298 | ind1B | 3210::IRGC 116950-1-1 | Sri Lanka |
| IRIS 313-8789 | aus | LALSAITA::IRGC 43915-1-1 | Bangladesh |
| IRIS 313-8568 | ind2 | LARHA MUGAD::IRGC 52339-1-1 | India |
| IRIS 313-12043 | ind3 | SAMBOK ANGKRANG::IRGC 81375-1 | Cambodia |
| IRIS 313-11049 | aus | AUS 219::IRGC 29031-1 | Bangladesh |
| IRIS 313-9111 | ind1A | XIA ZHI BAI::IRGC 53437-1-1 | China |
| IRIS 313-10497 | ind1A | CE IN TSAN::IRGC 4362-1 | China |
| IRIS 313-8697 | ind3 | YEBAWYIN::IRGC 33885-1-1 | Myanmar |
| IRIS 313-8727 | ind2 | T 315::IRGC 54792-1-1 | India |
| IRIS 313-10290 | ind1B | ELONI::IRGC 116980-1-1 | Surinam |
| IRIS 313-11745 | ind1A | AN FU ZHAN::IRGC 67878-1 | China |
| IRIS 313-9944 | indx | SOLOMON RED RICE::IRGC 50950-1-1 | Solomon Islands |
| IRIS 313-8980 | ind3 | E DAW HAWM::IRGC 47938-1-1 | Thailand |
| IRIS 313-8559 | ind2 | KEERIPALA CHILL PADDY::IRGC 49790-1-1 | India |
| IRIS 313-10337 | indx | B 6149 F-MR-7::IRGC 117314-1-1 | Indonesia |
| IRIS 313-8854 | ind2 | CHAKOL::IRGC 77226-1-1 | Bangladesh |
| IRIS 313-9970 | ind2 | RACE PERUMAL::IRGC 55347-1-1 | Sri Lanka |
| IRIS 313-9400 | indx | NCS 964 C::IRGC 62604-1-1 | India |
| IRIS 313-11355 | indx | CN 44-40-7::IRGC 45368-1 | India |
| IRIS 313-10301 | indx | IRGA 959-1-2-2F-4-1-4A-6-CA-6X::IRGC 117006-1-1 | Brazil |
| IRIS 313-10178 | ind1A | GAO JIAO BAI::IRGC 68047-1-1 | China |
| IRIS 313-11164 | aus | BAK TULSI::IRGC 34831-1 | India |
| IRIS 313-9953 | indx | JHONA 101::IRGC 27976-1 | Mexico |
| IRIS 313-8386 | indx | ARC 10812::IRGC 21074-1-1 | India |
| IRIS 313-9917 | ind1B | CHANDINA::IRGC 36420-1-1 | Sri Lanka |
| IRIS 313-8492 | ind3 | KUNENG::IRGC 71545-1-1 | Malaysia |
| IRIS 313-8903 | ind3 | BPI 76 NON SENSITIVE (GREEN)::IRGC 9790-1-1 | Philippines |
| IRIS 313-8725 | ind3 | PULUT BARAYA::IRGC 27393-1-1 | Indonesia |
| IRIS 313-10161 | ind1B | BR IRGA 409::IRGC 55915-1-1 | Brazil |
| IRIS 313-8796 | ind2 | DUDH KADAR::IRGC 67707-1-1 | India |
| IRIS 313-7690 | indx | IR 2344-P1 PB-9-3-2B::IRGC 39317-C1-G1 | Philippines |
| IRIS 313-10690 | ind3 | TELURIKAN::IRGC 27478-1 | Indonesia |
| IRIS 313-10480 | indx | PULUT NANGKA 16::IRGC 3630-1 | Indonesia |
| IRIS 313-8571 | ind3 | ES 21::IRGC 56171-1-1 | Tanzania |
| IRIS 313-10307 | indx | INIAP 6::IRGC 117002-1-1 | Ecuador |
| IRIS 313-11057 | aus | AUS 308::IRGC 29096-1 | Bangladesh |
| IRIS 313-8982 | indx | ARC 18112::IRGC 42274-1-1 | India |
| IRIS 313-10527 | ind2 | ADT 12::IRGC 6254-1 | India |
| IRIS 313-9989 | ind3 | MELEKE::IRGC 56823-1-1 | Ivory Coast |
| IRIS 313-8986 | indx | ARC 10754::IRGC 12603-1-1 | India |
| IRIS 313-8585 | indx | ARC 11901::IRGC 21727-1-1 | India |
| IRIS 313-11326 | indx | BR 51-115-4::IRGC 43999-1 | Bangladesh |
| IRIS 313-10394 | indx | IR 80310-12-B-1-3-B::IRGC 117307-1-1 | Philippines |
| IRIS 313-9139 | ind2 | GOJOL GORIA::IRGC 26629-1-1 | Bangladesh |
| IRIS 313-9065 | ind1A | PAI CHUEH CHIU LIU::IRGC 34259-1-1 | China |
| IRIS 313-9922 | ind1B | IRI 339::IRGC 46956-1-1 | South Korea |
| IRIS 313-9505 | ind1A | KORASISI::IRGC 5285-1-1 | Philippines |
| IRIS 313-11138 | ind3 | NGASEIN THEEDAT (C 30)::IRGC 33504-1 | Myanmar |
| IRIS 313-10392 | ind1B | IR 77390-1-6-4-19-1-B::IRGC 117303-1-1 | Philippines |
| IRIS 313-8244 | ind2 | POKKALI::IRGC 8948-1-1 | Sri Lanka |
| IRIS 313-10041 | trop | BOTOHAVANA MENA::IRGC 69349-1-1 | Madagascar |
| IRIS 313-9262 | ind2 | JHODI BIRUN::IRGC 31812-1-1 | Bangladesh |
| IRIS 313-9841 | indx | PULU RENNI::IRGC 27386-1 | Surinam |
| IRIS 313-11044 | trop | ARC 18502::IRGC 51749-2 | Malaysia |
| IRIS 313-10035 | ind2 | RIZ TYPE SORGHO::IRGC 69015-1-1 | Madagascar |
| IRIS 313-11555 | indx | PA WOON::IRGC 58656-1 | Sierra Leone |
| IRIS 313-10385 | ind1B | IR 75870-5-8-5-B-1::IRGC 117297-1-1 | Philippines |
| IRIS 313-11921 | ind3 | KHAO GAHB BUA::IRGC 74937-1 | Thailand |
| IRIS 313-9115 | ind3 | LEUANG YAI 29-12-2::IRGC 881-1-1 | Thailand |
| IRIS 313-11052 | aus | AUS 278::IRGC 29068-1 | Bangladesh |
| IRIS 313-10652 | ind3 | CHAO PEUAK DENG::IRGC 11602-1 | Laos |
| IRIS 313-9227 | ind1B | WP 65::IRGC 36526-1-1 | Thailand |
| IRIS 313-11140 | ind3 | PADAN::IRGC 33544-1 | Myanmar |
| IRIS 313-11058 | aus | AUS 329::IRGC 29116-1 | Bangladesh |
| IRIS 313-8743 | indx | NIAO YAO::IRGC 5496-1-1 | Taiwan |
| IRIS 313-10000 | ind1B | SUWEON 311::IRGC 61890-1-1 | South Korea |
| IRIS 313-10374 | indx | IR 69502-6-SRN-3-UBN-1-B::IRGC 117290-1-1 | Philippines |
| IRIS 313-10768 | ind3 | GADABUNG (GUNDIL)::IRGC 17569-1 | Indonesia |
| IRIS 313-8645 | ind1A | PAI YI PING::IRGC 1368-1-1 | China |
| IRIS 313-10268 | indx | FONAIAP 2::IRGC 116985-1-1 | Venezuela |
| IRIS 313-11010 | ind3 | PULUT PUTIH::IRGC 27426-1 | Indonesia |
| IRIS 313-9433 | ind2 | GOKULGANJA::IRGC 45701-1-1 | India |
