# Supplementary material for: New insights into QTNs and potential candidate genes governing rice yield via a multi-model genome-wide association study
Source: BMC Plant Biol. 2024 Feb 20;24:124. doi: 10.1186/s12870-024-04810-5 (PMC10877931; doi:10.1186/s12870-024-04810-5)
Supplement: Supplementary file 5 — Supplementary material 5. [file 12870_2024_4810_MOESM5_ESM.docx]

| **Trait** | **Mean ± SE^a^** | **Median^b^** | **Min^c^** | **Max^d^** | **Range^e^** | **Skewness** | **Kurtosis** |
| --- | --- | --- | --- | --- | --- | --- | --- |
| PlHt | 127.25 ± 1.81 | 129.90 | 72.10 | 187.90 | 115.80 | -0.17 | -0.84 |
| DTF | 98.380 ± 1.01 | 97.50 | 59.00 | 132.00 | 73.00 | 0.03 | -0.07 |
| PYD | 2333.19 ± 92.36 | 2163.75 | 213.75 | 5399.17 | 5185.42 | 0.44 | -0.71 |

**Table S2:** Summary statistics of three rice grain yield-related traits recorded on selected accessions.

Pl Ht, Plant height; DTF, Days to flowering; PYD, Plot yield

^a^Mean ± standard error of the observed trait values.

^b^Median observed trait value among accessions.

^c^Minimum observed trait value among accessions.

^d^Maximum observed trait value among accessions.

^e^Range, difference between maximum and minimum observed trait value.
