# Supplementary material for: New insights into QTNs and potential candidate genes governing rice yield via a multi-model genome-wide association study
Source: BMC Plant Biol. 2024 Feb 20;24:124. doi: 10.1186/s12870-024-04810-5 (PMC10877931; doi:10.1186/s12870-024-04810-5)
Supplement: Supplementary file 6 — Supplementary material 6. [file 12870_2024_4810_MOESM6_ESM.docx]

| Table S3 Summary of the different genetic diversity parameters used to infer the extent of genetic variability available in selected subset of rice accessions. | | | | | | | | | |
| --- | --- | --- | --- | --- | --- | --- | --- | --- | --- |
| **Marker diversity** | **Minimum** | **Maximum** | **Mean** |  |  |  |  |  |  |
| PiperBP | 0.05 | 0.5 | 0.28551 |  |  |  |  |  |  |
| ThetaperBP | 0.09 | 0.38 | 0.23 |  |  |  |  |  |  |
| Average Minor Allele Frequency | 0.05 | 0.5 | 0.2 |  |  |  |  |  |  |
| H_o_ | 0.001 | 0.26 | 0.016 |  |  |  |  |  |  |
| Average Major allele Frequency | 0.5 | 0.948 | 0.747 |  |  |  |  |  |  |

PiPerBP, nucleotide diversity per base pair/ Genetic diversity;

ThetaPerBP, the expected number of polymorphic sites per nucleotide;

H_o, observed heterozygosity_
